# Directly Administered Antiretroviral Therapy for HIV-Infected Individuals in Opioid Treatment Programs: Results from a Randomized Clinical Trial

**DOI:** 10.1371/journal.pone.0068286

**Published:** 2013-07-16

**Authors:** Gregory M. Lucas, Bernadette Anna Mullen, Noya Galai, Richard D. Moore, Katie Cook, Mary E. McCaul, Sheldon Glass, Krisann K. Oursler, Cynthia Rand

**Affiliations:** 1 Division of Infectious Diseases, Department of Medicine, Johns Hopkins University School of Medicine, Baltimore, Maryland, United States of America; 2 Department of Epidemiology, Johns Hopkins University Bloomberg School of Public Health, Baltimore, Maryland and the Department of Statistics, University of Haifa, Haifa, Israel; 3 Division of General Internal Medicine, Department of Medicine, Johns Hopkins University School of Medicine, Baltimore, Maryland, United States of America; 4 Division of Pulmonary and Critical Care, Department of Medicine, Johns Hopkins University School of Medicine, Baltimore, Maryland, United States of America; 5 Department of Psychiatry and Behavioral Sciences, Johns Hopkins University School of Medicine, Baltimore, Maryland, United States of America; 6 Division of Gerontology and Geriatric Medicine, Department of Medicine, University of Maryland School of Medicine and the Department of Veterans Affairs Medical Center, Baltimore, Maryland, United States of America; Indiana University and Moi University, United States of America

## Abstract

**Background:**

Data regarding the efficacy of directly administered antiretroviral therapy (DAART) are mixed. Opioid treatment programs (OTPs) provide a convenient framework for DAART. In a randomized controlled trial, we compared DAART and self-administered therapy (SAT) among HIV-infected subjects attending five OTPs in Baltimore, MD.

**Methods:**

HIV-infected individuals attending OTPs were eligible if they were not taking antiretroviral therapy (ART) or were virologically failing ART at last clinical assessment. In subjects assigned to DAART, we observed one ART dose per weekday at the OTP for up to 12 months. SAT subjects administered ART at home. The primary efficacy comparison was the between-arm difference in the average proportions with HIV RNA <50 copies/mL during the intervention phase (3-, 6-, and 12-month study visits), using a logistic regression model accounting for intra-person correlation due to repeated observations. Adherence was measured with electronic monitors in both arms.

**Results:**

We randomized 55 and 52 subjects from five Baltimore OTPs to DAART and SAT, respectively. The average proportions with HIV RNA <50 copies/mL during the intervention phase were 0.51 in DAART and 0.40 in SAT (difference 0.11, 95% CI: −0.020 to 0.24). There were no significant differences between arms in electronically-measured adherence, average CD4 cell increase from baseline, average change in log_10_ HIV RNA from baseline, opportunistic conditions, hospitalizations, mortality, or the development of new drug resistance mutations.

**Conclusions:**

In this randomized trial, we found little evidence that DAART provided clinical benefits compared to SAT among HIV-infected subjects attending OTPs.

**Trial Registration:**

ClinicalTrails.gov NCT00279110 NCT00279110&quest;term&hairsp;&equals;&hairsp;NCT00279110&amp;rank&hairsp;&equals;&hairsp;1

## Introduction

Studies evaluating the efficacy of directly administered antiretroviral therapy (DAART) for the treatment of HIV-infected individuals have yielded mixed results. A systematic review and meta-analysis of randomized trials by Ford and colleagues found no evidence overall for DAART benefit [1]. However, both the Ford analysis and a second systematic review [2], which included both randomized and non-randomized comparative studies, provided evidence of DAART efficacy when targeted to patient groups at high-risk for non-adherence, particularly substance abusers [3]–[5], and no evidence of efficacy when used in unselected or antiretroviral-naïve patient populations [6], [7].

Opioid treatment programs (OTPs) provide a potential framework for DAART because patients visit multiple times each week to receive opioid agonist medication. Moreover, maintenance treatment at OTPs permits prolonged DAART, which is difficult in models based on outreach workers. Based on promising results from a non-randomized pilot study [8], we conducted a randomized trial to evaluate the efficacy of DAART in OTPs.

## Methods

### Design and Follow-up

We conducted a randomized controlled trial comparing 12 months of DAART to self-administered therapy (SAT) in five Baltimore OTPs - 3 hospital associated (1 Veterans Administration) and 2 free-standing - which has been described previously [9]. Participants completed study visits at baseline, 3, 6, 12, and 18 months (6 months after the conclusion of the intervention). All visits included an interviewer-administered survey, blood sampling for HIV RNA and CD4 cell measurements, and urine for drug testing. Plasma was stored at a central repository for resistance testing.

We monitored adherence with electronic pill bottle monitors (MEMS 6 TrackCap, AARDEX Group, Ltd., Sion, Switzerland) for the first 2 months after subjects started antiretroviral therapy (ART). We selected a single medication for monitoring according to the following hierarchy: dosed most frequently, combination preparation, protease inhibitor, and non-nucleoside reverse transcriptase inhibitor. We gave all participants an electronic monitor for use at home. Additionally, we used separate monitors to record OTP doses for participants assigned to the DAART arm. We instructed participants on the purpose and use of the monitors and asked them to return at 1 week, 4 weeks, and 8 weeks to download data from the monitors. Research assistants did not review recorded adherence data with participants. The protocol for this trial and supporting CONSORT checklist are available as supporting information; see Checklist S1 and Protocol S1.

### Ethics Statement

Each participant provided written informed consent and the study protocol was approved by the Johns Hopkins Medicine Institutional Review Board (IRB), the University of Maryland IRB, and the Veterans Administration Research and Development Committee. This trial was registered at ClinicalTrials.gov (NCT00279110).

### Eligibility and Randomization

Individuals were eligible for the study if they were 18 years of age or older, HIV seropositive, not taking ART or virologically failing therapy, and had received methadone or buprenorphine for ≥3 weeks at the OTP with no plans to discontinue. We also required verbal approval from participants’ HIV providers and confirmation of active insurance coverage for ART. Exclusion criteria included ART dosed more frequently than twice daily, use of liquid medication, and use of a regimen that was predicted to have fewer than 1.5 active drugs (based on published interpretative guidelines [10]) according to previous genotypic resistance tests (if available).

To prevent knowledge of treatment assignment from influencing the selection of antiretroviral drugs, we required HIV providers to prescribe ART regimens prior to randomization. We used a commercial statistical software package to generate random treatment assignments to DAART and SAT in a 1∶1 ratio, in randomly varied block sizes between 2 and 8, stratified by study site and ART exposure at baseline (naïve or experienced). The treatment assignment list was incorporated into a Microsoft Access-based program that revealed individual assignments sequentially as new participants were enrolled. The study assignment list was concealed from study staff in a password-protected file.

### Interventions

Two pharmacies packaged medications for the DAART arm in single-dose plastic bags that were labeled with medication and dosing information. When participants attended the OTP for methadone or buprenorphine they went to a private office where a research assistant or a methadone nurse observed them take ART doses. The dose observer at each site encouraged DAART subjects to maintain adherence with self-administered doses and helped participants trouble-shoot difficulties that they were having with ART or other medical issues, but did not deliver a manual-based behavioral intervention directed at improving adherence. We gave participants take-home ART doses for weekends and (if applicable) weekdays that they were not required to attend the OTP. Participants who were taking twice-daily regimens self-administered evening doses. We provided DAART subjects with emergency doses to take if they missed a scheduled OTP visit. Participants assigned to SAT self-administered all ART doses. SAT participants were free to engage in adherence services offered by their HIV clinics or providers.

### Outcomes

The study’s primary outcome was HIV RNA <50 copies/mL. Specifically, we compared the average proportion of subjects in each arm with HIV RNA <50 copies/mL during the intervention phase (3-, 6- and 12-month study visits) using a generalized estimating equation (GEE) to account for intra-subject correlation. Electronic adherence measures included overall adherence (recorded doses divided by expected doses in the monitoring period), proportions adherent above thresholds of 80% and 95%, and proportion with a 72-hour period without a recorded dose. Secondary outcomes included HIV RNA <400 copies/mL, change in log_10_ HIV RNA, change in CD4 cell count, and acquisition of new drug resistance mutations during the intervention phase. We also measured HIV RNA and CD4 count differences at 18 months (6 months after the intervention phase) to assess the persistence of any intervention effects. Other secondary outcomes include retention to the OTP, urine drug tests positive for opiates or cocaine, self-reported emergency department use and hospitalizations, new opportunistic diseases [11] (excluding recurrent bacterial pneumonia) that were confirmed by medical record review, and mortality.

We assessed the acquisition of new drug resistance mutations by conducting genotypic resistance tests (Quest Diagnostics, Chantilly, VA) on paired failure and baseline samples. Failure samples were defined as the latest sample from the 3, 6, or 12 month visits in which the HIV RNA was ≥500 copies/mL. We defined new drug resistance mutations as clinically significant drug mutations listed in the International AIDS Society compendium [10] that were detected in the failure sample but not in the baseline sample.

### Power Calculations and Statistical Methods

Based on our pilot experience [8], we estimated that a 20% increase in the average HIV RNA suppression rate was feasible with DAART compared to SAT and represented a clinically relevant benefit for a relatively intensive adherence intervention [12]. We initially planned to compare HIV RNA suppression rates at a single time point, but revised our analytic approach and power calculations in response to challenges meeting a larger recruitment target [9]. Using a repeated measures analytic approach, we calculated that a sample size of 120 provided ≥83% power to detect a true average viral suppression difference of 20% between arms assuming: (a) a 2-sided type 1 error rate of 0.05; (b) 15% loss-to-follow-up; (c) an intra-subject constant correlation between 3 repeated follow-up measures of 0.2, and (d) an average viral suppression rate between 25% and 40% in the less effective arm. Power calculations were performed with PASS 11 software (NCSS, LLC, Kaysville, Utah) procedure for comparing two proportions in a repeated measures design.

Analyses were based on the intent-to-treat approach. For the primary analysis and other binary outcomes, we evaluated the difference between arms using a logistic regression model with two explanatory variables: an indicator for follow-up versus baseline visits (X1 = 0 for baseline, X1 = 1 for visits at 3, 6, and 12 months), and an interaction term between the intervention indicator (X2 = 0 for SAT, X2 = 1 for DAART) and the follow-up indicator (X3 = X1*X2, which takes on the value of 1 for DAART on follow-up and 0 otherwise). Note that the intervention indicator, X2, was not included in the main model considering the randomized design of the trial, which tends to balance both measured and unmeasured covariates. However, since this was a small trial we ran additional models in sensitivity analyses that adjusted for baseline values of log_10_ HIV RNA. For the main two-factor model, the coefficient X1 represented the difference in the odds of viral suppression between the intervention phase and baseline in the SAT arm, while the coefficient X3 represented the difference between DAART and SAT in the odds of viral suppression during the intervention phase versus baseline. The model accounted for the intra-person dependency of observations using the generalized estimating equation (GEE) approach [13] for parameter estimation and assuming a uniform correlation between any pair of repeated observations in the same person. The GEE approach provides valid and robust estimates for model parameters and their standard errors for longitudinal data. We used model output to estimate average proportions achieving viral suppression during the intervention phase in the two arms, with effect size shown as differences in proportions with 95% confidence intervals (CI). In the primary analysis, missing viral load data were ignored. However, we also conducted a secondary analysis in which missing viral load data were considered to be failures of viral suppression.

For continuous outcomes (log_10_ HIV RNA level and CD4 cell counts), we used linear regression GEE models to compare the average levels between the two treatment arms over follow-up, while accounting for the repeated measures design. These models included the same two factors, X1 and X3, described above. Undetectable HIV RNA values were assigned a value equal to the limits of assay detection (50 copies/mL). Estimated differences and 95% CI were based on model outputs. We compared retention to OTPs between arms using a Cox proportional hazards model for time from baseline to exit from the OTP, with results reported as a hazard ratio (HR) with 95% CI. Additional secondary endpoints measured at a single point in time were evaluated as differences in means for continuous outcomes or differences in proportions for binary outcomes. The estimated differences with 95% CI were calculated by using two-sample t-tests for means or Fischer’s exact test for proportions. In post-hoc analyses, we assessed the associations of selected factors with HIV RNA <50 copies/mL during the intervention phase with GEE logistic regression models as described above, with results reported as odds ratios (OR) and 95% CI. We assessed potential effect modification between these factors and the association between study arm and viral load suppression by combining terms in models and reporting P values for interaction terms. STATA version 12 software (StataCorp, LP, College Station, Texas) was used for statistical analyses.

## Results

### Participant Disposition and Characteristics

**Figure 1:**
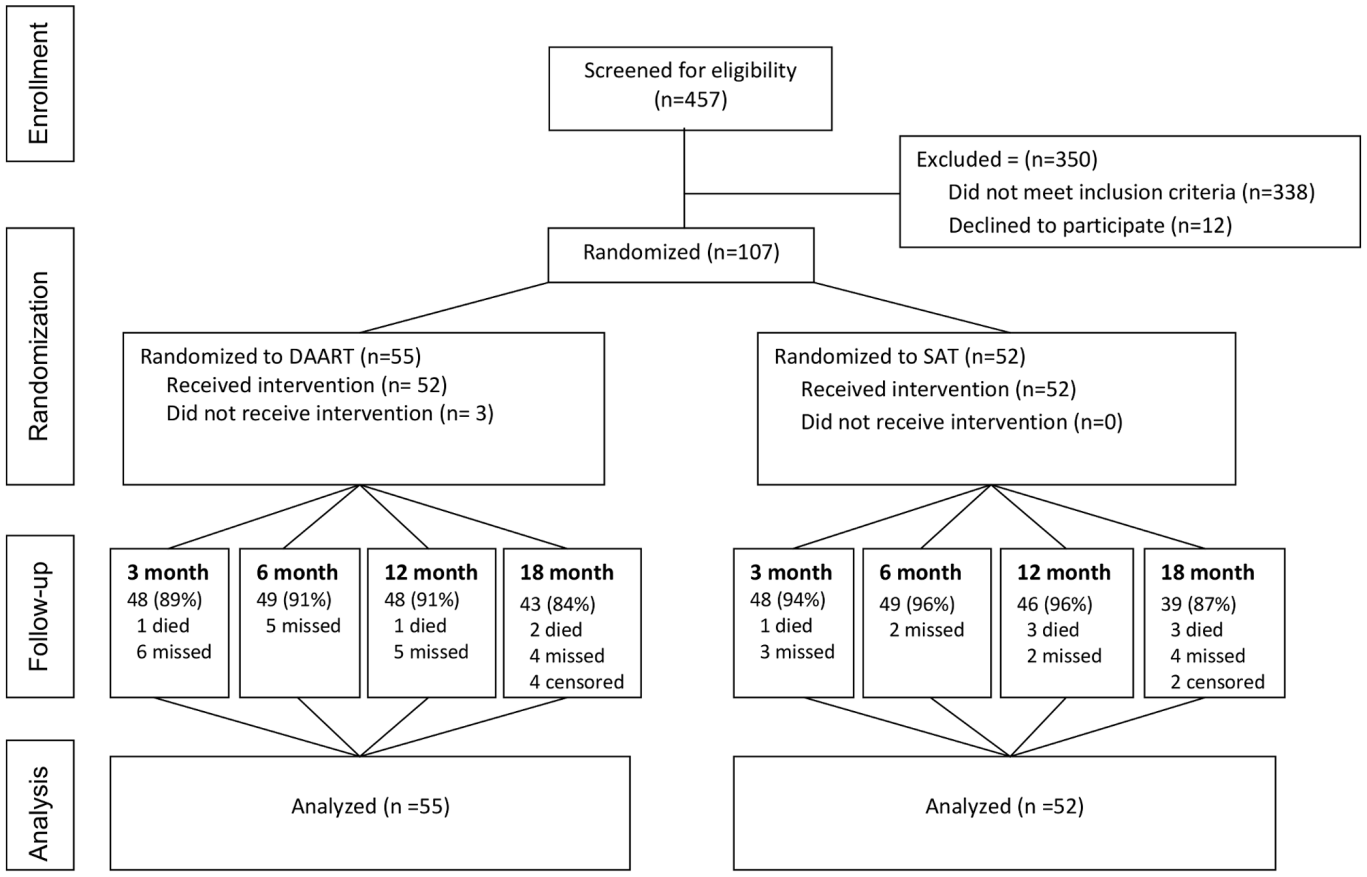
Study screening, enrollment, and disposition. Censored subjects had not reached the indicated study visit prior to the administrative closure of the study. DAART, directly administered antiretroviral therapy; SAT, self-administered therapy.

We screened 457 individuals and enrolled 107 subjects between May 2006 and May 2010, with 55 assigned to DAART and 52 to SAT (Figure 1). Three subjects assigned to DAART did not receive any observed doses. The overall study visit completion rate (excluding deaths) was 93% during the intervention phase (3–12 months) and was similar in the two arms.

Participant demographics, substance abuse history, and HIV-related characteristics were similar in the study arms (Table 1). Subjects had received methadone or buprenorphine for a median of 11 months at baseline, and approximately one-third was ART naïve. Compared to DAART participants, subjects assigned to SAT had somewhat lower CD4 cell counts and higher HIV RNA at baseline (Table 1). Three subjects assigned to DAART and none assigned to SAT had HIV RNA <50 copies/mL at the baseline study visit. Most participants used ritonavir-boosted protease inhibitors and a majority used regimens that were dosed once daily, with similar ART selection in the study arms.

**Table 1:** Baseline characteristics of HIV-infected participants in a randomized trial comparing directly administered antiretroviral therapy with self-administered therapy in opioid treatment programs, Baltimore, Maryland, 2006–2010.

Characteristic	SAT (N = 52)	DAART (N = 55)
***Demographic and psychosocial***		
Opioid treatment program (enrollment site), n (%)		
1 (hospital-based)	19 (37)	20 (36)
2 (free-standing)	17 (33)	18 (33)
3 (hospital-based)	4 (8)	4 (7)
4 (free-standing)	7 (13)	7 (13)
5 (hospital-based)	5 (10)	6 (11)
Female, n (%)	27 (52)	24 (44)
Race, n (%)		
Black	44 (85)	44 (80)
White/other	8 (15)	11 (20)
Age, years, median (IQR)	47 (42–52)	47 (41–51)
High school graduate or equivalent, n (%)	29 (56)	27 (49)
Homeless, self-described, n (%)	20 (39)	12 (23)
Receiving public assistance or social security disability, n (%)	41 (79)	50 (91)
Employed, n (%)	8 (15)	8 (15)
CES-D short-form score^a^, median (IQR)	11 (5–16)	11 (6–15)
***Substance abuse related***		
Duration in opioid treatment program, months, median (IQR)	10 (2–44)	11 (2–51)
Receiving methadone, n (%)	48 (92)	51 (93)
Methadone daily dose, mg, median (IQR)	95 (70–120)	80 (70–110)
Receiving buprenorphine, n (%)	4 (8)	4 (7)
Buprenorphine dose, mg, median (IQR)	19 (12–26)	21 (10–30)
Dosed at opioid treatment program ≥5 days per week, n (%)	43 (83)	44 (80)
History of drug injection, n (%)		
Never injected	8 (15)	2 (4)
Last injected >6 months ago	18 (35)	31 (56)
Injected within last 6 months	26 (50)	22 (40)
Urine drug test, positive results, n (%)		
Opiate	9 (17)	12 (22)
Cocaine	18 (35)	24 (44)
AUDIT b score ≥8, n (%)	10 (19)	10 (18)
***HIV related***		
Emergency department visit or hospitalization in prior 3 months, n (%)	25 (48)	27 (49)
Prior antiretroviral exposure, n (%)		
Naïve	13 (25)	20 (37)
Exposure to ≤2 drug classes	31 (60)	28 (51)
Exposure to ≥3 drug classed	8 (15)	7 (13)
Hepatitis C co-infected	45 (88)	49 (91)
Nadir CD4 count, cells/mm^3^, median (IQR)	108 (22–202)	89 (46–229)
Current CD4 count, cells/mm^3^, median (IQR)	154 (70–282)	244 (70–361)
Current HIV RNA, log_10_ copies/mL, median (IQR)	4.7 (4.2–5.1)	4.6 (3.7–5.0)
HIV RNA <400 copies/mL, n (%)	2 (4)	7 (13)
HIV RNA <50 copies/mL, n (%)	0 (0)	3 (5)
Category of prescribed antiretroviral regimen, n (%)		
PI+NRTIs	39 (75)	44 (80)
NNRTI+NRTIs	7 (13)	9 (16)
Other	6 (12)	2 (4)
Drug classes included in prescribed regimen, n (%)		
NRTI	48 (92)	49 (89)
Ritonavir-boosted PI	41 (79)	44 (80)
PI (not boosted with ritonavir)	3 (6)	1 (2)
NNRTI	10 (19)	11 (20)
Integrase inhibitor	5 (10)	2 (4)
Dosing frequency of prescribed regimen, n (%)		
Once daily	29 (56)	36 (65)
Twice daily	23 (44)	19 (35)

SAT, self-administered therapy; DAART, directly administered therapy; PI, protease inhibitor; NRTI, nucleoside reverse transcriptase inhibitor; NNRTI, non-nucleoside reverse transcriptase inhibitor.

a Center for Epidemiologic Studies Depression (CES-D) short form scale [14]. Higher values indicate more numerous or severe depressive symptoms (range 0 to 30).

b Alcohol Use Disorders Identification Test (AUDIT) score [15] of 8 or more is associated with hazardous drinking.

### Intervention Delivery

DAART was intended to be provided for 12 months. Among the 55 subjects assigned to DAART, the median time retained to the intervention was 267 days (interquartile range [IQR] 113–357). Research assistants observed a median of 90 doses (IQR 28–183) in DAART participants, and this varied by participants’ OTP reporting frequency at baseline (medians of 43 and 134 observed doses in those reporting to the OTP<5 days and ≥5 days per week, respectively). Fifty six percent (IQR 20%–79%) of expected observed doses were actually observed. Drop-out from the OTP or ART discontinuation accounted for a substantial proportion of non-observed doses. For example, when considering only the weeks in which DAART participants were retained to the intervention, 72% (IQR 56%–83%) of expected observed doses were observed. During electronic adherence monitoring in the first 2 months of the intervention, a median of 53% (IQR 33%–63%) of all doses taken by subjects in the DAART arm was observed in an OTP.

### Primary Viral Load Outcome

**Figure 2:**
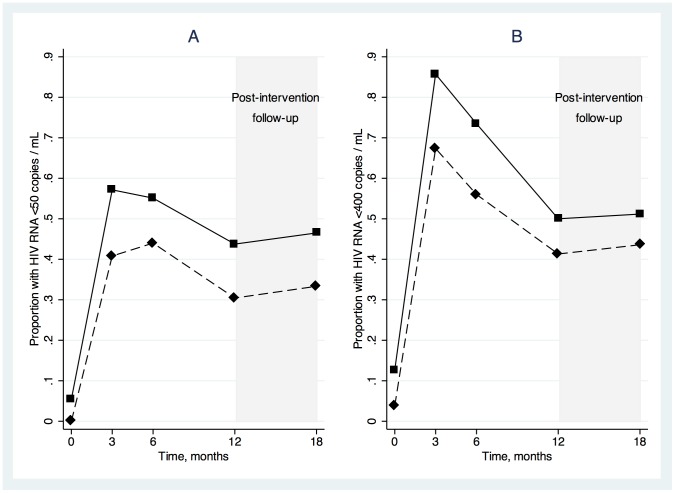
HIV RNA suppression, at cutoffs of <50 (A) and <400 copies/mL (B), at baseline, 3-, 6-, 12-, and 18-month follow-up visits, stratified by study arm, with missing values ignored. Directly administered antiretroviral therapy (DAART) is shown with square markers and solid lines and self-administered therapy (SAT) is shown with diamond markers and dashed lines.

The proportions of SAT and DAART subjects with HIV RNA <50 copies/mL at each visit are shown in Figure 2A. The primary outcome was the GEE model-estimated average proportion of participants with HIV RNA <50 copies/mL during the active intervention period (months 3 through 12). In the primary analysis, where missing data were ignored, the average proportions with HIV RNA <50 copies/mL during the intervention phase were 0.51 in DAART and 0.40 in SAT (difference, 0.11; 95% CI, −0.02 to 0.24)(Table 2). In the secondary analysis, in which missing values were considered failures, the model-estimated average proportions with HIV RNA <50 copies/mL during the intervention phase were 0.45 in DAART and 0.37 in SAT (difference, 0.08; 95% CI, −0.04 to 0.20).

**Table 2:** Study outcomes in a randomized trial comparing directly administered antiretroviral therapy with self-administered therapy in opioid treatment programs, Baltimore, Maryland, 2006–2010.

	SAT (N = 52)	DAART (N = 55)	Difference^a^ (95% CI)	P value
**Virologic and immunologic outcomes**				
Average proportion with HIV RNA <50 copies/mL during intervention^b^	0.40	0.51	0.11 (−0.02, 0.24)	0.087
Average proportion with HIV RNA <400 copies/mL during intervention	0.56	0.67	0.11 (−0.02, 0.24)	0.10
Average change from baseline in log_10_ HIV RNAduring intervention	−1.46	−1.68	−0.22 (−0.56, 0.12)	0.21
Average change from baseline in CD4 cell counts during intervention	45	78	32 (−11, 75)	0.15
**Electronic adherence monitoring** c				
Adherence^d^, mean	71%	75%	4% (−5% to 13%)	0.41
Adherence ≥80%, n (proportion)	25 (0.49)	28 (0.54)	0.05 (−0.14 to 0.24)	0.70
Adherence ≥95%, n (proportion)	7 (0.14)	7 (0.14)	0 (−0.13 to 0.13)	1.0
≥72-hour period without dose, n (proportion)	29 (0.57)	27 (0.52)	−0.04 (−0.24 to 0.14)	0.69
**Clinical events, n (proportion)**				
≥1emergency department visit	35 (0.67)	35 (0.64)	−0.04 (−0.22 to 0.14)	0.84
≥1 hospitalization	26 (0.50)	29 (0.53)	0.03 (−0.16 to 0.22)	0.85
≥1 opportunistic disease	7 (0.13)	5 (0.09)	−0.04 (−0.16 to 0.08)	0.55
Mortality	7 (0.13)	4 (0.07)	−0.06 (−0.18 to 0.05)	0.35

SAT, self-administered therapy; DAART, directly administered therapy; CI, confidence interval.

a Difference in proportions (binary outcomes) or means (continuous outcomes) between DAART and SAT.

b Primary outcome.

c Electronic adherence data available for 51 and 52 SAT and DAART participants, respectively.

d Adherence is (doses recorded/doses expected in the monitoring period)*100.

### Other Viral Load and CD4 Count Outcomes

**Figure 3:**
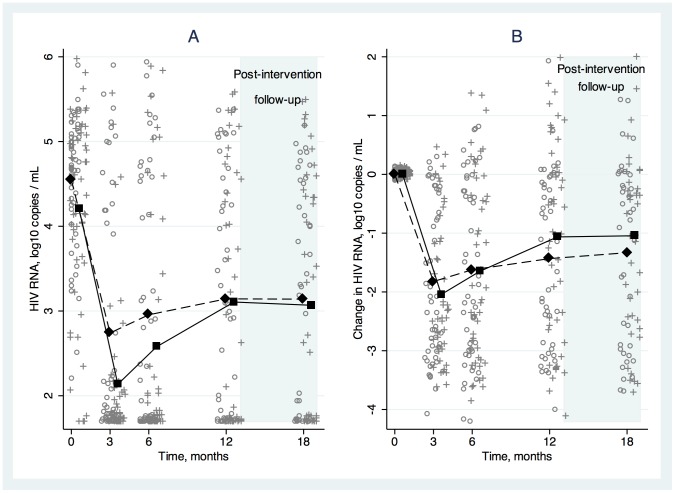
Average log_10_ HIV RNA (A) and change from baseline in log_10_ HIV RNA (B) over time, stratified by study arm. Directly administered antiretroviral therapy (DAART) is shown with square markers and solid lines and self-administered therapy (SAT) is shown with diamond markers and dashed lines. Individual-level data are shown at each time point (o indicating SAT and+indicating DAART).

**Figure 4:**
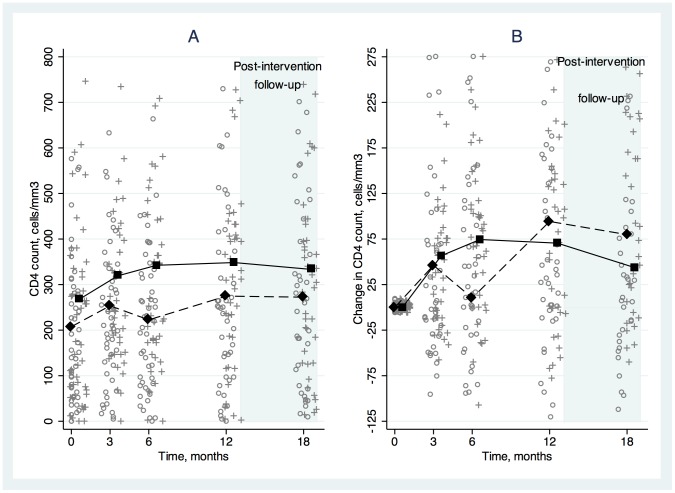
Average CD4 cell count (A) and change from baseline in CD4 cell count (B) over time, stratified by study arm. Directly administered antiretroviral therapy (DAART) is shown with square markers and solid lines and self-administered therapy (SAT) is shown with diamond markers and dashed lines. Individual-level data are shown at each time point (o indicating SAT and+indicating DAART).

The proportions of SAT and DAART subjects with HIV RNA <400 copies/mL at each visit are shown in Figure 2B. The model-estimated average proportion with HIV RNA <400 copies/mL during the intervention phase was higher in the DAART than the SAT arm, although the difference was not statistically significant (Table 2). Figures 3 and 4 show average values and average changes from baseline at each study time point by treatment arm for log_10_ HIV RNA and CD4 cell counts, respectively. The differences between arms in log_10_ HIV RNA and CD4 cell changes from baseline were not statistically significant (Table 2).

At 18 months (6 months following the intervention phase) there were no significant differences between DAART and SAT in the proportions with HIV RNA <50 copies/mL (0.47 vs. 0.33; difference, 0.13; 95% CI, −0.08 to 0.34), the proportions with HIV RNA <400 copies/mL (0.51 vs. 0.44; difference, 0.08; 95% CI, −0.14 to 0.29), average change in HIV RNA from baseline (−1.04 vs. −1.33 log_10_ copies/mL; difference, 0.29; 95% CI, −0.42 to 1.00), or average change in CD4 count from baseline (47 vs. 80 cells/mm^3^; difference, −33; 95% CI, −105 to 39).

### Electronic Adherence Monitoring, Clinical Outcomes and Drug Resistance

Electronic adherence data, covering the first 2 months of ART, were available from 51 of 52 SAT subjects and 52 of 55 DAART subjects. The median period of electronic monitoring was 57 days (IQR 55–61), with no difference by study arm (data not shown). Adherence was marginal: the average adherence was 73% and only 14% were ≥95% adherent. There were no statistically significant differences in any adherence parameters between the study arms (Table 2). The proportions of subjects with emergency department visits, hospitalizations, opportunistic diseases, or deaths during the intervention period were similar in the two arms (Table 2).

Fewer participants assigned to DAART (45%) than SAT (60%) met virologic failure criteria for resistance testing during the intervention phase, although the difference was not statistically significant (Table 3). Among those in whom resistance testing was done, the percentages with new drug resistance mutations were similar in DAART and SAT. The M184V and K103N mutations in reverse transcriptase were acquired most commonly. No subject acquired new protease inhibitor-associated mutations or mutations to more than one drug class.

**Table 3:** Virologic failure and acquisition of antiretroviral drug resistance mutations in a randomized trial comparing directly administered antiretroviral therapy with self-administered therapy in opioid treatment programs, Baltimore, Maryland, 2006–2010.

	SAT (N = 52)	DAART (N = 55)	P value
Virologic failure eligible for genotype testing	31/52 (60)	25/55 (45)	0.18
RNA from baseline and failure samples amplified	29/31 (94)	21/25 (84)	0.39
New drug resistance mutations detected	9/29 (31)	6/21 (29)	1.00
M184V	3	2	
K103N	3	3	
Protease mutations	0	0	
Mutations to >1 drug class	0	0	

SAT, self-administered therapy; DAART, directly administered therapy; CI, confidence interval.

### Substance Abuse Outcomes

The estimated OTP retention at 6 and 12 months were 78% and 60% in DAART, and 83% and 73% in SAT, respectively (HR for OTP discontinuation in DAART versus SAT, 1.3; 95% CI, 0.7–2.4). During the intervention phase, the average proportions with urine drug tests positive for opiates or cocaine were 0.33 and 0.37 in DAART and SAT, respectively (difference −0.04, 95% CI −0.18 to 0.10).

### Post-hoc Analyses

In post-hoc analyses, we examined the associations of selected baseline factors with the likelihood of achieving HIV RNA <50 copies/mL during the intervention phase in univariate models. Compared to ART-naïve subjects, ART-experienced subjects had similar odds of viral suppression (OR 0.98, 95% CI 0.56–1.72). Compared to subjects who had attended the OTP for >12 months at enrollment, those who has attended ≤12 months had lower odds of viral suppression (OR 0.58, 95% CI 0.34–0.98). Compared to subjects dosed at the OTP five or more days each week, those dosed less frequently than five days per week had lower odds of viral suppression (OR 0.55, 95% CI 0.28–1.08). There was no evidence that ART-exposure status, duration of OTP attendance, or OTP dosing frequency modified the association between study arm and HIV RNA <50 copies/mL during follow-up (range of P values for interactions 0.47–0.97, data not shown).

## Discussion

In this randomized trial of HIV-infected subjects receiving methadone or buprenorphine in OTPs, we found little evidence that DAART provided benefit compared to SAT. DAART was associated with a non-statistically significantly higher average proportion with HIV RNA <50 copies/mL during the intervention phase, the primary outcome. However, there were no differences between arms in average change in log_10_ HIV RNA or in CD4 cell counts and, importantly, there were no differences between arms in electronically measured adherence. OTP retention and urine drug test results for opiates or cocaine were also similar in the two arms. Few subjects acquired new drug resistance mutations during the intervention phase, and the proportions acquiring new drug resistance were similar in the study arms.

Notably, our failure to find a benefit with DAART did not occur in the context of a high overall success rate as has been the case in some DAART trials that enrolled individuals at relatively low risk for virologic failure [6], [7] Rather, treatment outcomes were poor overall in our study, with only 14% having ≥95% measured adherence, less than 50% achieving HIV RNA <50 copies/mL on average, modest CD4 cell increases, and high rates of hospitalizations, opportunistic conditions, and mortality. In the DAART arm, only 56% of the expected observed doses were actually observed due to non-retention to the intervention (e.g., drop-out from the OTP or ART discontinuation) or missed OTP visits. This low delivery of the intervention may explain its absence of efficacy.

Our findings should be interpreted in the context of other randomized trials of DAART in similar populations. In contrast to our findings, three prior randomized trials among substance abusers reported statistically significantly higher rates of viral suppression compared with DAART compared to SAT, and two of the three trials also reported statistically significantly larger increases in CD4 cell counts with DAART [3]–[5]. Two of these trials [3], [5] used outreach-based DAART (community health workers and a health van) and are difficult to compare directly to our model. However, similar to our study, a trial conducted by Berg and colleagues compared DAART to SAT among HIV-infected subjects attending OTPs in the Bronx, New York [4]. In the Bronx study, the rate of viral suppression was 0.27 higher in the DAART arm than the SAT arm at 24 weeks [16], compared to average differences in HIV RNA <50 copies/mL of 0.11 (ignoring missing data) and 0.08 (considering missing values to be failure) in our trial. Moreover, in the Bronx trial, objectively measured adherence rates were statistically significantly higher in the DAART arm compared to the SAT arm [4], whereas we found no difference in electronically monitored adherence.

There are potential explanations for the different findings of our trial and the Bronx trial. First, subjects enrolled in the Bronx study may have been more stable than those in our study. In the Bronx study, participants had been receiving methadone for a median of 10 years at enrollment, compared to a median of 11 months in our study. In a post-hoc analysis, we found that subjects who had attended the OTP for less than 1 year at study enrollment were significantly less likely to achieve viral load suppression than those who had attended the clinic for more than 1 year. Additionally, only 4% (3/77) subjects in the Bronx study left the OTP during the study, compared with 20% (21/107) in a similar time frame in our study. Second, the Bronx trial was conducted in a jointly administered network of OTPs that offered on-site medical and HIV care [17]. In contrast, our trial was conducted at 5 administratively distinct OTPs, only one of which offered on-site HIV treatment. The DAART intervention may have benefited from a greater degree of baseline service integration in the Bronx study. Finally, individuals who were dosed at the OTP less frequently than 5-times per week were ineligible for the Bronx study, whereas this was not an exclusion criterion in our study. Although only 19% of subjects in our study was dosed less frequently than 5-times weekly, this may have reduced the impact of the intervention for some subjects.

Despite success in some studies, it is difficult to formulate recommendations for DAART. Unlike tuberculosis, which is curable with 6 months of chemotherapy and where directly observed therapy has assumed central role, HIV treatment requires lifelong medication adherence. Existing research has not addressed the long-term effectiveness of DAART - most studies have used intervention periods of 6 months or less, and three studies where an early viral suppression benefit was observed reported that the differences between arms waned rapidly following intervention cessation [6], [16], [18].

Our study has limitations and strengths. First, our final sample size was small (107) and we failed to meet our revised enrollment target of 120. Consequently, results from our single trial cannot completely exclude a clinically significant difference in the study arms and our results should be interpreted in the context of other studies in this area. However, our failure to detect a statistically significant difference in viral suppression between the arms – the primary outcome - was supported by a cadre of secondary outcomes, including electronic adherence monitoring. Second, in response to lower than expected recruitment we changed our primary analysis from comparison of viral suppression at a single time point to a repeated measures analysis using GEE. While the repeated measures approach is robust and produces valid inferences, the comparison of average suppression rates may have less clinical relevance and less straightforward applicability to other work than comparison at a single time point.

Third, the median CD4 cell count at baseline was almost 100 cells/mm^3^ lower in the SAT arm than the DAART arm. Differences in baseline immune status and higher mortality in SAT compared to DAART may have affected the comparison of viral suppression rates. In the primary intent-to-treat analysis where missing values were ignored, higher mortality in the SAT arm may have reduced the apparent difference in viral suppression between the arms. In contrast, in the secondary intent-to-treat analysis where missing results were considered failures, differences in mortality or drop-out for other reasons would be reflected in viral suppression differences. In our analyses, the results were similar and inferences unchanged when missing values were ignored or treated as failures. Fourth, our study was conducted in Baltimore, Maryland, and may not reflect intervention efficacy in other domestic or international settings. The strengths of our study include >90% follow-up at study visits during the intervention phase, a 12-month intervention to assess longer-term efficacy, a rigorous electronic adherence monitoring protocol for both study arms, and a study assessment 6 months after the DAART conclusion to assesses post-intervention durability.

In conclusion, compared to SAT, we found that rates of viral suppression were similar with DAART or SAT among HIV-infected participants attending OTPs in Baltimore. There were no differences between the arms in electronically monitored adherence, CD4 cell count change, emergency room use, hospitalizations, retention to OTP, urine drug screen results, or acquired drug resistance.

## Supporting Information

Checklist S1 **CONSORT 2010 checklist of information to include when reporting a randomised trial.**
(DOC)

Protocol S1 **IRB-approved protocol for trial.**
(DOC)
