# Supplementary material for: Directly Administered Antiretroviral Therapy for HIV-Infected Individuals in Opioid Treatment Programs: Results from a Randomized Clinical Trial
Source: PLoS One. 2013 Jul 16;8(7):e68286. doi: 10.1371/journal.pone.0068286 (PMC3712961; doi:10.1371/journal.pone.0068286)
Supplement: Protocol S1 — IRB-approved protocol for trial. (DOC) [file pone.0068286.s002.doc]

1. **Protocol Title:** Directly administered vs. self-administered antiretroviral therapy in methadone clinics
2. **Protocol Version and Date of Version:** Version 2.5; June 17, 2010
3. **Study Summary**

We propose to conduct a randomized, unblinded, clinical trial of a medication adherence intervention in opioid-dependent, HIV-infected participants who are initiating new antiretroviral therapy, and who receive opioid agonist maintenance therapy with methadone or buprenorphine at opioid treatment programs (OTPs) in Baltimore, MD. Randomization will be stratified by study site and prior antiretroviral exposure. Two hundred participants will be randomly assigned 1:1 self-administered antiretroviral therapy (SAT) or directly administered antiretroviral therapy (DAART). Subjects assigned to DAART will take morning doses of antiretroviral therapy with a nurse or medical assistant in a private room at the OTP. DAART subjects will be transferred to self-administered therapy after 12 months. This is a 5 year study and participants will be enrolled between month 6 and month 42 of the study. The maximum follow-up for individual participants will be 18 months. Based on our pilot experience we anticipate 50% of subjects will be women, 80% African American, with a median age of 44 years. The following outcomes will be compared in the two study arms:

A. Suppression of the viral load (primary outcome),

B. Changes in CD4+ cell counts,

C. The development of antiretroviral drug resistance,

D. Retention to opioid agonist maintenance therapy, urine toxicology screens for drugs of abuse, and self-reported drug and alcohol use,

E. Self-reported adherence with therapy, retention to ART, and clinical and psychosocial moderators of adherence,

D. Electronically monitored medication adherence, using MEMS caps, in the first 2 months of the study

Outcomes data will be obtained at study assessment visits at baseline, 3 months, 6 months, 12 months, and 18 months. Participants will provide contact information, take an interviewer-administered survey, and provide blood and urine samples at study assessment visits. MEMS cap data will be captured at 1 month and 2 months. Subjects will be compensated for successful completion of study assessment visits and MEMS interrogations.

1. **Study Procedures**
2. Study design, including the sequence and timing of study procedures. Distinguish procedures that are experimental from those which are part of routine care.

Study Design

Recruitment and screening

• Inclusion / No exclusion criteria

• Verify coverage for ART costs

Contact medical provider

• Obtain verbal consent

• Medical provider selects antiretroviral regimen

Random treatment assignment

(stratify by prior ART exposure, treatment site, and HIV RNA)

SAT

(N =60)

DAART

(N =60)

Include in intent-to-treat analysis

Obtain written, informed consent

Study follow-up (0, 3, 6, 12, and 18 months)

1) HIV RNA, CD4+ cells, CBC, chemistry panel

2) Specimen repository for resistance tests

3) Urine toxicology screens

4) Interviewer-administered survey

5) MEMS monitoring (Specific aim 2)

Outcome Assessments

1) HIV RNA < 50 copies/ml (1° outcome)

2) Change in CD4+ cells

3) Development of new antiretroviral drug resistance

4) Substance abuse treatment outcomes

5) Adherence (MEMS) and psychobehavioral moderators of adherence

Study performance sites and IRB oversight

The proposed clinical trial will be conducted at opioid treatment programs (OTPs) in Baltimore.

• Program for Alcohol and Other Drug Dependencies (hereafter **PAODD**) – affiliated with Johns Hopkins; 911 N. Broadway, Baltimore, MD 21205.

Site PI: Gregory M. Lucas, MD

Director: Mary McCaul, PhD

• New Hope Treatment Center (hereafter **New Hope**) – affiliated with the Bon Secours Healthcare System (FWA00004991); 2401 W. Baltimore Street, Baltimore, MD 21223. Enrollment discontinued at this site on 6/20/2007 due to slow recruitment.

Site PI: Suyi Park, MD

Director: Carrie Little

• **Day Break** – affiliated with Glass Substance Abuse Programs, Inc. (FWA00004893); 2490 Giles Road, Baltimore, MD 21225.

Site PI: Sheldon Glass, MD

• **Man Alive**, Inc. (FWA00006062); 2117 Maryland Avenue, Baltimore, MD 21218. This site will be added to the study in August, 2007.

Site PI: Gregory M. Lucas, MD

Director: Karen Reese, MA, CAC-AD

• Baltimore Veterans Administration Substance Abuse Clinic (hereafter **VA**); 10 N. Greene Street, Baltimore, MD 21201.

Site PI: Kris Ann Oursler, MD

• Glass Substance Abuse Program (hereafter referred to as the Maryland General site), 821 N. Eutaw St., Suite 101 and 201, Baltimore, MD 21201 (FWA 00004893).

The New Hope, Day Break, Maryland General, and VA clinics will participate as sub-contractual sites, with previously agreed upon budgets. Neither the New Hope nor the Glass Substance Abuse Programs sites (Day Break or Maryland General sites) has an IRB or a mechanism for overseeing research in human subjects. Federalwide Assurances were obtained at each of these sites, giving research oversight to JH IRB2 (assurance number shown above). Research at the VA is overseen by both the University of Maryland IRB and by the VA Research and Development Committee. Approval from these two oversight bodies will be obtained separately. The demographic characteristics of clients at the performance sites are similar and are shown in the Table below.

| **Table. Cross-sectional comparison of methadone clinic study sites** | | | | | | |
| --- | --- | --- | --- | --- | --- | --- |
| **Factor** | **PAODD** | **New Hope** | **Day Break** | **Man Alive** | **VA** | **Maryland General** |
| **Clinic characteristics** |  |  |  |  |  |  |
| Current opiate agonist maintenance census, N | 153 | 450 | 1007 | 567 | 375 | 480 |
| HIV-infected, %* | 35% | 29% | 10% | 17% | 14% | 15% |
| Approximate number of HIV-infected individuals in clinic, N | 54 | 130 | 100 | 95 | 51 | 72 |
| Female | 42% | 35% | 55% | 35% | 2% | 40% |
| Age (years), median | 42 | 40 | 38 | 40 | 41 | 42 |
| African American, % | 88% | 90% | 84% | 85% | 91% | 87% |
| Methadone dose, average | 98 mg | 85 mg | 100 mg | 90 mg | 90 mg | 90 mg |
| Days per week methadone is dosed | 7 | 6 | 6 | 6 | 6 | 7 |
| 12-month methadone maintenance retention rate | 70% | 77% | 84% | 90% | 75% | 79% |
| Availability of on-site HIV counseling and testing | Yes | Yes | Yes | Yes | Yes | Yes |
| Availability of on-site primary HIV medical care | Yes | Yes | Yes | No | Yes | No |
| Methadone maintenance slots dedicated for HIV-infected persons (Ryan White Care Act) | Yes | Yes | No | Yes | N/A | No |

* Based on self-identification and anonymous HIV seroprevalence studies.

Inclusion criteria

1. Eighteen years of age or older,

2. Documented serologic evidence of HIV infection (positive ELISA and Western blot),

3. Identifiable medical provider, who is responsible for managing HIV treatment,

4. Proof that ART has been prescribed and that patient has prescription medication coverage,

5. Participant must have (at least one of the following)

a) A history ART treatment

b) Symptoms related to HIV infection

c) A nadir CD4+ cell count ≤ 350/mm^3^ or off-treatment HIV RNA > 55,000 copies/ml

6. Current plasma HIV RNA > 500 copies/ml,

7. Participant must be (at least one of the following)

a) Initiating ART for first time

b) Reinitiating prior therapy after stopping

c) Changing therapy due to virologic failure

d) Continuing therapy in the setting of viremia due to non-adherence

8. ART with ≥ 3 agents, including a protease inhibitor, a non-nucleoside reverse transcriptase inhibitor, or abacavir,

9. Methadone or buprenorphine maintenance therapy > 3 weeks, with no planned detoxification.

Exclusion criteria

1. Need to use ART dosed more frequently than twice daily,

2. Need to use a liquid preparation of antiretroviral medication,

3. Selected antiretroviral regimen has < 1.5 active drugs according to documented antiretroviral drug resistance (defined below),

4. Participation in another study or program that includes directly observed therapy.

5. Use of ART regimens that are expressly discouraged in DHHS HIV clinical care guidelines [1]

Inclusion criterion number 4 is designed to assure equal access to therapy at study enrollment. ART will not be provided as part of this study. Coverage for ART will be addressed through patients’ primary care clinics. In our study population, antiretroviral therapy coverage is generally obtained through Medicaid or the Maryland AIDS Drug Assistance Program (MADAP). Our site managers will assist individuals in accessing appropriate resources, but participants will not be enrolled until medication coverage and receipt of prescriptions from a medical provider has been verified. Exclusion criterion 3 is designed to exclude individuals with such extensive drug resistance that viral suppression is unlikely, even with high-level adherence. A summary resistance profile will be compiled that assumes that any resistance mutation or phenotypic drug resistance documented in the past is never lost. The cumulative mutation list will be entered into the Stanford University web-based HIV drug resistance database ([http://hivdb.stanford.edu](http://hivdb.stanford.edu/)) for analysis. Drugs in the proposed regimen will be considered to be fully active (i.e., score=1.0) if categorized as “susceptible” or “potential low-level resistance”, and to be partially active (i.e., score=0.5) if categorized as “low-level resistance” by the Stanford database. Drugs in the proposed regimen with “intermediate“ or “high-level” resistance will not be assumed to have any antiviral activity. Results from phenotypic resistance tests (if available) will be incorporated into the assessment individual drug activity. Enfuvirtide (fusion inhibitor) and raltegravir (integrase inhibitor) will be assumed to be fully active if they have not been used previously. CCR-5 inhibitors (e.g., maraviroc) will be assumed to be fully active if an agent in this drug class has not been used previously and a tropism assay performed within the previous 4 weeks shows exclusively R5-tropic virus. Scores form each drug in the proposed regimen will be summed for a “regimen activity score.” To be eligible for the study a participant’s “regimen activity score” must be greater than or equal to 1.5.

Enrollment and randomization

120 participants will be enrolled to assess the outcomes of this study.

Participants’ HIV medical providers will select the antiretroviral regimens to be used. Bias would be introduced if medical providers selected antiretroviral therapy regimens differently based upon knowledge of whether their patient had been randomized to DAART or to SAT. For example, it is possible that medical providers would be more likely to select an antiretroviral therapy regimen that is dosed once daily in individuals enrolled to DAART. This might lead to significant differences between the groups in the use of specific antiretroviral drugs or combinations that could confound the analysis. To avoid this potential source of bias, each medical provider will be asked to specify the particular antiretroviral regimen that he or she will prescribe prior to participant randomization. Our clinical trial is flexibly designed to assess the efficacy of DAART in patients with a range of clinical characteristics, and antiretroviral needs. The study coordinator will contact the data manager for random treatment assignment. Randomization (1:1; performed with Stata, version 8.0, College Station, TX), stratified by prior antiretroviral exposure (naïve or experienced), and study site (PAODD, New Hope, or Day Break), will be performed in blocks randomly varying sized 2 to 6. Antiretroviral naïve will be defined as having less than 4 weeks exposure to antiretroviral drugs.

DAART arm

Site managers will take verbal orders or receive facsimiles of prescriptions from participating medical providers. The site managers will transcribe the medication information to standardized pharmacy order forms, which specify how medications are to be packaged together. We have established a relationship with a single pharmacy, Johns Hopkins Pharmaquip, which will be used for all sites except the VA. Since veterans receive medications free of charge through the VA system, medications for this study will be prepared and packaged at the VA pharmacy. In accordance with pharmacy practice and guidelines, a licensed pharmacist will prepackage all doses of therapy in re-sealable plastic bags. Four-week supplies of therapy will be pre-packaged and will be delivered to the methadone clinics. Pre-packaged therapy will be stored in locked cabinets at each site. Participants will be given a 3-day “emergency supply” of their therapy to permit continued therapy on unplanned absences from the OTP. The site managers will replenish emergency supplies as needed.

Morning antiretroviral medications will be administered to participants on weekdays. Evening doses (when needed) and weekend doses will be self-administered. Antiretroviral medications will be dosed in a private office. In our pilot study, this was preferred by participants over receiving medications at the methadone dispensing window. Each of our three sites has a private office, which will be dedicated to antiretroviral therapy dosing, and medication and record storage.

At the time a participant receives a directly observed dose, he or she will also be given the appropriate number of individual doses for self-administration. Adherence with observed doses will be recorded on log sheets. Opiate-agonist maintenance treatment is dispensed 6 or 7 days a week at the participating clinics. When patients begin opiate-agonist therapy, they are required to come to the clinic every day that it is open to receive methadone or buprenorphine. As patients progress in their substance abuse recovery, they become eligible for take-home doses of agonist therapy, according to protocols that are based on achieving treatment goals. Take-home dosing of antiretroviral therapy will be modified over time to correspond with changes in opiate agonist therapy dosing. Site managers will provide unstructured feedback to HIV medical providers and communicate difficulties that patients may be having (e.g., side effects to therapy, frequent missed doses, etc.).

Participants assigned to DAART will receive this intervention for a maximum of 12 months. Participants or their medical providers may decide to discontinue antiretroviral therapy or DAART at any time. DAART will also be discontinued when patients leave the OTP. In this situation, participants may continue on antiretroviral therapy at the discretion of their medical providers. DAART participants who leave the OTP and subsequently return or who discontinue antiretroviral therapy temporarily (e.g., due to side effects) and subsequently restart will be permitted to restart DAART, providing that it is within 12 months of their original start date. Individuals will not be re-enrolled in the study. Following completion of DAART, participants will meet with the site manager to review their medications and develop a plan for maintaining adherence.

SAT arm

Subjects allocated to the SAT arm will self-administer antiretroviral therapy. Participants assigned to SAT will receive a level of support commensurate with standard clinical practice. Participants in SAT will not be precluded from participating in any adherence-promoting resources available in their clinic or the community. It is important to acknowledge that the DAART intervention is multifaceted, and its effects on adherence and treatment outcomes may be mediated through several mechanisms. Similarities and differences between DAART and SAT are shown in the Table below**.**

| Table. Comparison of interventional factors in DAART and SAT | | |
| --- | --- | --- |
| Factors | **DAART** | **SAT** |
| Medical follow-up by regular medical provider | √ | √ |
| ART chosen by medical provider prior to randomization | √ | √ |
| Proof of coverage for prescription medications prior to enrollment (access to therapy) | √ | √ |
| Access to adherence aids, interventions, or programs available through medical clinics or community resources (excluding other supervised therapy programs) | √ | √ |
| Study laboratory test results forwarded to medical providers to assist in patient management | √ | √ |
| Ingestion of ART supervised at OTP on weekday mornings | √ | No |
| Non-observed ART doses pre-packaged in re-sealable plastic bags | √ | No |
| Medication supplies monitored and refills obtained for participants by site managers | √ | No |
| Frequent contact with site managers - opportunity for dialogue/encouragement | √ | No |
| Facilitated communication with participants’ medical providers | √ | No |
| Medications for other medical and psychiatric conditions directly administered | No | No |

Participant follow-up tracking

In the proposed study we will obtain detailed contact information from participants, including current address, phone number, work number, cell phone number, and pager number. Additionally, we will ask participants to identify and provide contact information for two individuals with whom they have had longstanding relationships (such as family members). Prior to assessment visits, participants who remain in the OTPs will be given verbal reminders and a written reminder of their upcoming visit. Attempts will be made to locate study participants who are no longer attending the methadone clinic through reminder letters, telephone calls, and personal contacts.

Data collection timeline

| **Assessment/Procedure** | **Time (months)** | | | | | | |
| --- | --- | --- | --- | --- | --- | --- | --- |
|  | 0 | **1** | **2** | **3** | **6** | **12** | **18** |
| **Study assessment visits** |  |  |  |  |  |  |  |
| Demographic, contact, and clinical information | • |  |  |  |  |  |  |
| HIV-1 RNA level | • |  |  | • | • | • | • |
| CD4+ cell count | • |  |  | • | • | • | • |
| Plasma storage for resistance testing | • |  |  | • | • | • | • |
| Interviewer-administered survey | • |  |  | • | • | • | • |
| Urine toxicology screen | • |  |  | • | • | • | • |
| Reimbursement for assessment visit | $50 |  |  | $50 | $50 | $50 | $50 |
| **Electronic adherence monitoring** |  |  |  |  |  |  |  |
| Initiate MEMS monitoring period | • |  |  |  |  |  |  |
| MEMS caps interrogation |  | • | • |  |  |  |  |
| Reimbursement for MEMS sub-study |  | $100 total | |  |  |  |  |

Assessment visits

Study assessment visits will be performed by the study coordinator at the three performance sites. Each study participant will be assigned a unique identification number. This identification number will be used on each data form and clinical specimen to facilitate linkage of data. Names and other obvious identifiers will not be used on forms or clinical specimens. Data collection forms from our pilot study will be modified to create forms tailored for this randomized controlled trial. Study charts will be stored on-site at the methadone clinics in locked file cabinets. The data manager will enter information from paper forms into the electronic database. A programmer will design a Microsoft Access relational database, which will link data from the various sources by unique identifying number. Computers used for data entry will be password protected and data will be backed-up each night.

Clinical specimens (blood and urine samples) will be obtained by the study coordinator at assessment visits. In addition to the unique identification number, clinical specimens will be further identified by a two digit visit code, a one digit code for specimen type, and a one digit code identifying the specimen aliquot for banked plasma. Specimens will be transported back to Johns Hopkins in designated transport containers and processed the same day. Laboratory tests, including CD4 cell count and HIV RNA will be performed in real time, and test results will be sent to participants’ medical providers. Plasma for resistance tests will be processed and stored at -50° C for drug resistance testing. Paired resistance testing will be performed on the baseline sample and the last available sample in which the HIV RNA level was above 500 c/ml and the participant was taking antiretroviral therapy. Results of urine toxicology screens will be masked until the conclusion of the study, and then will only be available to study personnel conducting analyses. Results of the urine toxicology screens will not be made available at any time to medical providers, methadone clinic staff, or others without signed permission from the participant.

The questionnaire component of the assessment visits will be administered by the study coordinator by face-to-face interview at study assessment visits. The study coordinator will read questions to participants in a private office and record results directly onto a computer tablet using a Microsoft-Access-based data entry template.

Measurement methods, assays, and instruments

| **Factor assessed** | **Assay or instrument** |
| --- | --- |
| HIV-1 RNA | Amplicor 1.5 (Roche Diagnostics) |
| CD4+ cell count | Flow cytometry – Johns Hopkins Clinical Laboratory |
| Antiretroviral resistance | HIV-1 protease and reverse transcriptase genotyping (Quest Diagnostics, Baltimore, MD) |
| Substance use | Urine immunoassay for amphetamines, barbiturates, benzodiazepines, cocaine, opiates, cannabinoids, and phencyclidine (Friends Medical Laboratory, Inc., a CLIA-approved facility [http://www.friendslab.com](http://www.friendslab.com/)) |
| Self-reported drug and alcohol use | ACASI-based survey – modified questions from the Addiction Severity Index (ASI); AUDIT survey for at-risk alcohol use. |
| Self-reported adherence | ACASI-based survey – 4 questions. |
| Depression/anxiety | ACASI-based survey – Hospital anxiety and depression scale (HADS) |
| Quality of life | ACASI-based survey - MOS-HIV Health Survey |
| Objectively measured adherence | • MEMS VI TrackCaps, hardware and software (Aardex Corp. Union City, CA)  • Supervised doses in the DAART arm logged by the site managers |

Electronic adherence monitoring (MEMS caps)

Study participants will undergo electronic adherence monitoring for a 2-month period at study enrollment using a modified version of a technique described by Wall and associates [2]. A single medication will be selected for electronic monitoring for each participant according to the following hierarchy of criteria: 1) medication dosed most frequently, 2) combination preparations, containing two or more antiretroviral agents, 3) medication with the lowest number of pills per dose. The study coordinators will instruct the participants on the purpose and proper use of the MEMS caps at an instruction visits when participant initiate their HIV medications. The site managers will be available to all participants for any questions regarding use of the MEMS caps during the monitoring period. Participants will bring their MEMS cap bottles into the methadone clinic at 1.5 weeks, 4 weeks, and 8 weeks, and will be reimbursed for their efforts at these times. MEMS data will be downloaded to a computerized database using hardware and software provided by the manufacturer (Aardex, Union City, CA). Site managers will remind participants (DAART and SAT) when their bottles should be brought to clinic for interrogation.

**SAT group**: Subjects assigned to SAT will bring their medications into the methadone clinic at study initiation. The site managers will review medication dosing directions and the proper use of the MEMS caps with all SAT subjects. For each subject, the medication that has been selected for monitoring will be placed in a MEMS bottle and subjects will be instructed to open and close the bottle for each dose that they take, and not to take out more than one dose at a time. If participants require a refill of their monitored medication during a monitoring period, they will bring the new medication to the methadone clinic and the site manager will refill the monitored bottle. SAT participants who prefer to use a medication pill box (< 10% based on our prior experience) will be asked to keep the MEMS bottle by their pill box, and to open and close it each time they take a dose from the pill box. Subjects who use the MEMS caps in this way will be labeled to assess for heterogeneity in the adherence data. Financial incentives will be offered at baseline, 1.5 weeks, 4 weeks, and 8-weeks to optimize the return of MEMS units for interrogation and minimize missing data.

**DAART group**: In the DAART arm, electronic adherence monitoring will be adapted to the protocol for supervised dosing. There are two potential ways that this adaptation might be accomplished. First, single MEMS caps might be used for both supervised and unsupervised dosing for each DAART participant. In this case participants would be required to bring their MEMS bottles into the methadone clinic with them each week day. The advantage of this approach is that it most closely approximates how adherence will be monitored in the SAT group. Wall et al.[2] used this approach in a study of supervised versus unsupervised zidovudine therapy in HIV-infected DUs in a methadone clinic (reviewed in **Section B.3.** of the grant proposal). They reported that participants forgot to bring their monitored medication bottle with them to the methadone clinic an average of two out of every five days, leading to problems with underestimated adherence in the supervised therapy group. For this reason we have selected a modified approach, whereby each participant will use one MEMS bottle for supervised doses at the methadone clinic (e.g., weekday mornings) and a second MEMS bottle for unsupervised doses (e.g., nights and weekends). For each participant, the medication that has been selected for adherence monitoring will be removed from dose packs and apportioned to the MEMS bottle to be used the methadone clinic and the MEMS bottle to be used in the participant’s home. When a participant is given a supervised dose in the methadone clinic, the site manager will give him or her non-monitored medications from a dose pack and remove the monitored medication from the participant’s on-site MEMS bottle. When a participant takes an unsupervised dose (e.g., in the evening), he or she will take the monitored medication from the MEMS bottle and other medications from his or her regular prepackaged dose pack. Using this modified approach, adherence will be electronically monitored in both the DAART and SAT groups. Financial incentives will be offered at baseline, 1.5 weeks, 4 weeks, and 8 weeks to optimize the return of MEMS units for interrogation and minimize missing data.

**MEMS pilot study**

To gain experience with MEMS hardware and software, and to troubleshoot and optimize our MEMS monitoring protocol for the main study, we will conduct a MEMS pilot study 2 to 6 participants, the details of which are presented below.

Goals:

- Gain experience with the MEMS monitoring hardware
- Gain experience with the MEMS monitoring software and data storage programs
- Gain experience with the instructing subjects on the proper use of MEMS caps
- Gain experience with the quality assurance instruments and techniques that supplement the use of MEMS monitors.
- Troubleshoot logistical problems that may arise with MEMS monitoring in the main study

Study site: The main study will be conducted at three methadone clinics in Baltimore. However, the MEMS pilot will be conducted at only one of these sites, the Johns-Hopkins-associated Program for Alcoholism and Other Drug Dependencies (PAODD), located at 911 N. Broadway.

Study personnel: No new personnel will be used in this pilot study

Participants: Two to six (2-6) participants for the MEMS pilot study will be recruited subjects who are HIV-infected and receiving methadone or buprenorphine maintenance therapy at the PAODD. We will recruit subjects from an HIV primary-care satellite clinic located at the PAODD. We will ask a doctor or nurse to present the study to eligible individuals to seek volunteers. No printed recruiting material will be used.

Enrollment criteria:

Inclusion criteria

- HIV-infected
- Receiving methadone or buprenorphine therapy
- Receiving antiretroviral therapy
- Competent and willing to provide written informed consent

Exclusion criteria

- Non-English speaking
- Need to use a pill-box

MEMS Pilot protocol:

- Participants will bring their antiretroviral medications to the PAODD
- Participants will have one (1) of their antiretroviral drugs selected for MEMS monitoring using the selection criteria that will be used in the main study.
- Participants will be assigned a 3-digit identification number for this study and all MEMS adherence information will be collected and linked to this number only. No personal identifiers will be used to link adherence data to participants.
- The bottle cap of the medication selected for monitoring will be replaced with a MEMS 6 Track Cap (Aardex Ltd., Zug, Switzerland).
- Participants will be instructed on the purpose and proper use of MEMS caps using an established instruction protocol (enclosed)
- Participants will use the MEMS monitor for 1 week and return the bottle cap for data download using the MEMS communicator. Participants will be debriefed using a standarized instrument (protocol) and open-ended questions. Participants will be compensated $10 for returning the MEMS cap for this visit.
- Participants will use the MEMS monitor for a second week and return the bottle cap for data download and debriefing as described above. Participants will be compensated $10 for returning the MEMS cap for this visit.
- Participants will use the MEMS monitor for an additional two (2) weeks and return the device for a final data download and debriefing. Participants will be compensated $20 for returning the MEMS cap visit.
- Adherence data will be reviewed on-screen with participants if they wish.
- Adherence data and experiences will be reviewed in aggregate with Dr. Cynthia Rand (co-investigator and adherence expert) to optimize MEMS protocol for main study

Total duration of MEMS pilot study: 6 months

Total duration of participant follow-up: 4 weeks

Number of visits: 5; screening, baseline instruction visit, 1-week follow-up, 2-week follow-up, 4-week follow-up.

Compensation: $40 for adherence with all study visits

1. Study duration and number of study visits required of research subjects.

This will be a 5-year research study, with active subject enrollment from month 6 to month 42. Individual participants will be followed for up to 18 months. The active DAART intervention will last 12 months, with subjects switched to self-administered therapy thereafter. Participants will be request to attend 5 assessment visits and to use MEMS caps for the first 2 months and return them for interrogation at months 1 and 2, for a total of 7 study-related visits (Table).

| **Assessment/Procedure** | **Time (months)** | | | | | | |
| --- | --- | --- | --- | --- | --- | --- | --- |
|  | 0 | **1** | **2** | **3** | **6** | **12** | **18** |
| **Study assessment visits** |  |  |  |  |  |  |  |
| Demographic, contact, and clinical information | • |  |  |  |  |  |  |
| HIV-1 RNA level | • |  |  | • | • | • | • |
| CD4+ cell count | • |  |  | • | • | • | • |
| Plasma storage for resistance testing | • |  |  | • | • | • | • |
| ACASI-based survey | • |  |  | • | • | • | • |
| Urine toxicology screen | • |  |  | • | • | • | • |
| Reimbursement for assessment visit | $50 |  |  | $50 | $50 | $50 | $50 |
| **Electronic adherence monitoring** |  |  |  |  |  |  |  |
| Initiate MEMS monitoring period | • |  |  |  |  |  |  |

**MEMS pilot study**

Total duration of MEMS pilot study: 6 months

Total duration of participant follow-up: 4 weeks

Number of visits: 5; screening, baseline instruction visit, 1-week follow-up, 2-week follow-up, 4-week follow-up.

1. Blinding including justification for blinding or not blinding the trial, if applicable.

This trial will not be blinded. Blinding the participants, their medical providers, or study staff is not possible in this comparative trial of an adherence intervention.

1. Justification on why subjects will not receive standard care or will have current therapy stopped.

N/A

1. Justification for inclusion of a placebo or non-treatment group.

N/A

1. Definition of treatment failure or subject removal criteria.

Subjects may be removed form the study if their continued participation poses a risk to themselves or other, if a participant withdraws consent, or if a participants’ medical provider withdraws consent.

1. Description of what happens to subjects with therapy when study ends or if a subject’s participation in the study is ended prematurely.

All subjects receiving DAART (the active intervention) will be switched to self-administered therapy at 12 months and followed through 18-months. Any participant who leaves the intervention prior to this will be given any remaining antiretroviral medications that have been pre-packaged and may continue to take medication on a self-administered basis as determined by his or her primary HIV care provider.

1. **Study Subjects**
2. The subject population including how subjects will be identified and recruited.

Participants will be recruited through patient self-referral, referral from addictions counselors, and referral from medical providers. The DAART program will be presented initially to all known HIV-infected persons by their substance abuse counselor or their medical provider, and individuals expressing interest will be referred to the site managers. Site-specific study recruitment flyers will also be posted at each site. Full-time site managers will be available at the study sites for recruitment. Eligible subjects must provide signed, informed consent to participate in the study. Non-identifying demographic data, whether or not enrolled in the study, and the reason for failure to enroll if not enrolled (e.g., failure to meet inclusion and exclusion criteria) will be recorded in all individuals screened for the study. This will permit us to quantify the reasons why referred subjects were not eligible for enrollment, the reasons why eligible individuals declined to participate, and to compare characteristics of joiners and non-joiners. Based on our pilot experience we anticipate 50% of subjects will be women, 80% African American, with a median age of 44 years.

**Screening**. Inclusion and exclusion criteria (listed below) will be assessed for each subject screened for the study. In most cases, we anticipate that study eligibility will be determined on the basis of subject interview and review of medical records. In some cases (e.g., absence of recent laboratory monitoring) we may ask participants to allow us to draw a blood sample to measure HIV RNA, CD4 cell count, and/or an HIV resistance test in order to determine if they meet inclusion criteria 5 and 6, and do not meet exclusion criterion 3.

**Recruitment of participants for MEMS pilot study**

We will recruit subjects from an HIV primary-care satellite clinic located at the PAODD. We will ask a doctor or nurse to present the study to eligible individuals to seek volunteers. No printed recruiting material will be used.

1. Inclusion criteria.

1. Eighteen years of age or older,

2. Documented serologic evidence of HIV infection (positive ELISA and Western blot),

3. Identifiable medical provider, who is responsible for managing HIV treatment,

4. Proof that ART has been prescribed and that patient has prescription medication coverage,

5. Participant must have (at least one of the following)

a) A history ART treatment

b) Symptoms related to HIV infection

c) A nadir CD4+ cell count ≤ 350/mm^3^ or off-treatment HIV RNA > 55,000 copies/ml

6. Current plasma HIV RNA > 500 copies/ml,

7. Participant must be (at least one of the following)

a) Initiating ART for first time

b) Reinitiating prior therapy after stopping

c) Changing therapy due to virologic failure

d) Continuing therapy in the setting of viremia due to non-adherence

8. ART with ≥ 3 agents, including a protease inhibitor, a non-nucleoside reverse transcriptase inhibitor, or abacavir,

9. Methadone or buprenorphine maintenance therapy > 3 weeks, with no planned detoxification.

1. Exclusion criteria.

1. Need to use ART dosed more frequently than twice daily,

2. Need to use a liquid preparation of antiretroviral medication,

3. Selected antiretroviral regimen has < 1.5 active drugs according to documented antiretroviral drug resistance (defined below),

4. Participation in another study or program that includes directly observed therapy.

5. Use of ART regimens that are expressly discouraged in DHHS HIV clinical care guidelines [1]

Inclusion criterion number 4 is designed to assure equal access to therapy at study enrollment. ART will not be provided as part of this study. Coverage for ART will be addressed through patients’ primary care clinics. In our study population, antiretroviral therapy coverage is generally obtained through Medicaid or the Maryland AIDS Drug Assistance Program (MADAP). Our site managers will assist individuals in accessing appropriate resources, but participants will not be enrolled until medication coverage and receipt of prescriptions from a medical provider has been verified. Exclusion criterion 3 is designed to exclude individuals with such extensive drug resistance that viral suppression is unlikely, even with high-level adherence. A summary resistance profile will be compiled that assumes that any resistance mutation or phenotypic drug resistance documented in the past is never lost. The cumulative mutation list will be entered into the Stanford University web-based HIV drug resistance database ([http://hivdb.stanford.edu](http://hivdb.stanford.edu/)) for analysis. Drugs in the proposed regimen will be considered to be fully active (i.e., score=1.0) if categorized as “susceptible” or “potential low-level resistance”, and to be partially active (i.e., score=0.5) if categorized as “low-level resistance” by the Stanford database. Drugs in the proposed regimen with “intermediate“ or “high-level” resistance will not be assumed to have any antiviral activity. Results from phenotypic resistance tests (if available) will be incorporated into the assessment individual drug activity. Enfuvirtide (fusion inhibitor) and raltegravir (integrase inhibitor) will be assumed to be fully active if they have not been used previously. CCR-5 inhibitors (e.g., maraviroc) will be assumed to be fully active if an agent in this drug class has not been used previously and a tropism assay performed within the previous 4 weeks shows exclusively R5-tropic virus. Scores form each drug in the proposed regimen will be summed for a “regimen activity score.” To be eligible for the study a participant’s “regimen activity score” must be greater than or equal to 1.5.

1. If research involves study of existing samples/records, describe how authorization to access samples/records will be obtained.

During informed consent, participants will be asked to sign two release-of-information forms, one for HIV-related medical records, and one for substance abuse treatment records (Appendix).

**Inclusion and exclusion criteria for MEMS pilot study**

Inclusion criteria

- HIV-infected
- Receiving methadone or buprenorphine therapy
- Receiving antiretroviral therapy
- Competent and willing to provide written informed consent

Exclusion criteria

- Non-English speaking
- Need to use a pill-box

1. **Drugs/Substance/Devices**
2. The rationale for choosing the drug or substance dose or for choosing the device to be used.

N/A

1. For IND/IDE studies, a summary of preclinical and early human studies.

N/A

1. Justification and safety information if FDA approved drugs will be administered for non-FDA approved indications or if doses or routes of administration or subject populations are changed.

N/A

1. Justification and safety information if non-FDA approved drugs without an IND# will be administered

N/A

1. **Study Statistics**
2. Primary outcome variable

The primary endpoint for this study, suppression of the viral load below 50 c/ml at the 3 follow-up visits during the active intervention period (3, 6 and 12 months), will be compared between the two study arms using statistical methods for repeated observations. The analytical approach will be intent-to-treat, with missing values excluded.

1. Secondary outcome variables and analysis

| **Outcome** | **Outcome type &**  **comparison** | **Analytical approach** | **Statistical methods** |
| --- | --- | --- | --- |
| **Viral suppression** | | | |
| HIV RNA < 50 c/mL;  HIV RNA < 400 c/mL | Binary: Assess whether any treatment group effect observed during active intervention phase (0-12 months) remains at 6 months post intervention (18 months post enrollment) | Intent-to-treat, *missing values excluded* | Chi-square for each time point and logistic model with GEE for overall comparison; assesses for interaction between study arm and study phase |
| HIV RNA < 50 c/mL;  HIV RNA < 400 c/mL | Binary: Assess mechanisms by which DAART may be efficacious by comparing primary analysis (where missing values are excluded) to alternative analytical approaches. | - Intent-to-treat, *missing values considered failure* - *On treatment analysis (time points where subjects not taking ART excluded)* | Chi-square for each time point and logistic model with GEE for overall comparison |
| Change in HIV RNA (log_10_) from baseline | Continuous: compare in DAART and SAT at 4 time points (3, 6, 12, and 18 months) | Intent-to-treat, missing values excluded | ANOVA for each time point and linear regression with GEE for overall comparison |
| **CD4 cell recovery** | | | |
| Change in CD4 cell count from baseline | Continuous: compare in DAART and SAT during active intervention (3, 6, and 12 months) | Intent-to-treat | ANOVA for each time point and linear regression with GEE for overall comparison |
| CD4 cell count | Continuous | Intent-to-treat | Linear regression with GEE & adjusting for initial CD4 at baseline |
| **Antiretroviral therapy use** | | | |
| Retention to ART | Time to failure: first day of ART cessation lasting ≥ 14 days, self-report | Intent-to-treat | Kaplan-Meier curves and Log-rank test |
| Cumulative ART use time | Continuous: number of weeks ART used in 12 months, self-report | Intent-to-treat | Wilcoxon rank-sum test |
| **Development of drug resistance** | | | |
| Antiretroviral drug resistance | Binary: presence of IAS-defined drug resistance mutations (during 12-month intervention phase) that were not present in baseline sample or in resistance test conducted prior to study (if available) | Intent-to-treat, missing values excluded | Chi-square test |
| **Substance abuse treatment outcomes** | | | |
| Retention to substance abuse treatment | Time to failure: discontinuation from opioid-treatment program | Intent-to-treat | Kaplan-Meier with log-rank test; Cox proportional hazards model adjusting for prior time in methadone program |
| Active drug use | Continuous: proportion of total available drug screens positive for opiates or cocaine. [Protocol-driven and clinical drug screens (required monthly by law in methadone clinics)] | Intent-to-treat, missing excluded, censored at methadone discontinuation | Wilcoxon rank-sum; Poisson regression with count of positive screens per follow-up months excluding periods with no testing |
| Electronically monitored adherence | MEMS cap data | i) overall adherence: (number of bottle openings) ÷ (number of prescribed doses) in monitoring period  ii) breakdown of overall adherence into a) weekday morning adherence and b) night and weekend adherence  iii) Proportion of subjects with a drug holiday (≥ 48 hours without recorded dose).  iv) Proportion of days with correct dosing, defined as the number of days in which the  correct number of bottle openings were recorded (separated by ≥ 8 hours if twice daily dosing) divided by the number of days in the monitoring period  v) Proportion of days with at least one dose, defined as the number of days with ≥ 1 dose divided by the number of days in the monitoring period | Chi-square, ANOVA or non-parametric techniques as appropriate |
| **Clinical outcomes** | | | |
| Clinical disease progression | Time to event: development of new CDC category C condition or death | Intent-to-treat | Kaplan-Meier curves and Log rank test |

1. Statistical plan including sample size justification and interim data analysis.

We will carry out a primary unadjusted analysis comparing study endpoints in the DAART and SAT arms. Two-sided P values < 0.05 will be considered to be statistically significant. The primary endpoint for this study will be suppression of the viral load below 50 c/ml evaluated at the 3 follow-up visits during the active intervention period (3, 6 and 12 months). Statistical generalized linear models for repeated observations will be used with the generalized estimating equations (GEE) approach to account for intra-person correlation. An intent-to-treat rule will be used, with missing values excluded. An 18-month follow-up (6 months after DAART intervention concludes) will be performed as a secondary analysis to assess durability. A supplementary on-treatment analysis will also be conducted. Secondary, multivariate analyses will be used to adjust for differences in possible confounding variables that may arise despite randomization. Data collection for the study will include detailed demographic, clinical, and psychosocial factors that may be associated with study outcomes, including prior opportunistic conditions, hepatitis B and C, baseline CD4 count and HIV RNA level, prior treatment experience, and validated metrics of drug and alcohol abuse, depression, and quality of life. We will assess whether adjustment for potential confounders, using multivariate modelling techniques, affects study inferences. For the analysis of immune restoration, we will use an intention-to-treat approach, with last observation carried forward for missing data points. The mean change in CD4+ cell count from baseline will be compared in the study arms at the designated follow-up times. Alternatively, a survival analysis approach will be used with time until first CD4 increase >100 or counts >500 as the outcome and censoring at last visit for each person.

The development of new antiretroviral drug resistance will be compared in the DAART and SAT arms. Genotypic antiretroviral resistance testing will be performed on the last available plasma sample from (3, 6, 12, or 18 months) when the HIV RNA > 500 c/ml and the subject remains on antiretroviral therapy. Stored plasma from baseline will be run simultaneously to determine if new resistance mutations have developed. Antiretroviral resistance testing is generally not possible at viral loads below 500 c/ml to 1,000 c/ml. Drug resistance mutations will be interpreted according to the classification system of the International AIDS Society – USA [3]. Development of antiretroviral resistance will be defined as the presence of ≥ 1 major resistance mutation in the protease or reverse transcriptase genes during study follow-up that was not present at baseline. Subjects with HIV RNA levels < 500 c/ml at all follow-up time points will be considered to have not developed drug resistance. Participants who are lost to follow-up, or who have HIV RNA levels > 500 c/ml, but in whom genotypic antiretroviral resistance testing is not technically feasible, will be omitted from the analysis. Exploratory analyses are planned on treatment-naïve and –experienced subgroups, and on differences in the development of protease inhibitor-, non-nucleoside reverse transcriptase inhibitor-, and nucleoside reverse transcriptase inhibitor-associated resistance mutations.

Retention to opiate agonist therapy will be compared in the DAART and SAT arms using survival analysis techniques. Follow-up time will be modeled from the date participants initiated opiate agonist therapy (origin), with subjects coming under observation at study enrollment, to account for delayed entry and potential lead time bias. Subjects will be considered to have failed when they discontinue or are discharged from methadone maintenance therapy. We will also compare the proportion of participants with active substance abuse at baseline and during the study in the DAART and SAT groups. Active substance abuse will be defined as having a urine toxicology screen positive for drugs of abuse or self-reported (ACASI) illicit drug use, or at-risk alcohol use in the 3 months preceding the survey assessment. Logistic regression with generalized estimating equations, will be used to compare the longitudinal risk of active substance abuse in the study groups, with standard errors adjusted for repeated observations.

Adherence with antiretroviral therapy will be evaluated by both self-report (interview) and by electronic monitors. Self-reported adherence will be analyzed as a dichotomous variable (< or ≥ 2 missed doses in the 2 weeks preceding study assessment), as we have previously found this cutpoint to be associated with viral suppression in subjects receiving ART [13]. Electronically-monitored adherence will be analyzed in several ways.

An overall adherence score for the 2-month monitoring period will be calculated as:

{[no. of recorded doses] / [no. of prescribed doses]} X 100

For each 24-hour period, bottle opening events in excess of the prescribed number of doses will not be counted. As an exploratory analysis, we will compare weekday morning adherence and night and weekend adherence separately in the study arms. Additionally, we will compare the following domains in DAART and SAT: drug holidays, defined as the absence of a recorded dose in a time period ≥ 48 hours, the proportion of days with correct dosing, defined as the number of days in which the correct number of bottle openings were recorded (separated by ≥ 8 hours if twice daily dosing) divided by the number of days in the monitoring period, and the proportion of days with at least one dose, defined as the number of days with ≥ 1 dose divided by the number of days in the monitoring period. The latter domain is sometimes used to avoid underestimation of adherence that may occur when participants remove more than one dose from the bottle at a single opening (i.e., “pocket doses.”)[112]. Analysis of variance or non-parametric techniques will be used to compare the average adherence in the DAART and SAT groups. Participants who are lost to follow-up within 2 months of study enrollment or who fail to provide any electronic adherence data for other reasons will be categorized separately. We will conduct sensitivity analyses to determine how extreme the missing data must be to compromise the substantive findings of the adherence comparison between the study arms.

**Statistical power**

In this analysis, we will use methods that can incorporate repeated measurements and account for possible intra-person correlations. Such methods include generalized linear models with generalized estimating equation (GEE) approach or random effects models that can handle binary as well as continuous outcomes. Thus, overall, the number of observations available for comparison of the experimental and control arms of the study will be increased and missing observations will be excluded only for the time points where values of the outcome are missing. Calculations of sample sizes are based on models for longitudinal data analysis (Diggle PJ, Heagerty P, Liang KY, Zeger SL. Analysis of Longitudinal Data. 2002 Oxford University Press, Publ.) are shown in the following table.

The table below shows the power to detect a range of deltas in the DAART arm according to different viral suppression rates in the SAT arm, assuming alpha=0.05, enrollment of 60 per study arm, an intra-subject correlation of 0.2, and 15% loss to follow-up over one year. The table demonstrates >80% to detect deltas >0.20 across varying assumptions.

| **Table. Study Power with sample size of 60 per arm.** | | | | |
| --- | --- | --- | --- | --- |
| **Proportion in control (P0)** | **Delta in DAART arm (P1-P0)** | | | |
|  | **0.15** | **0.18** | **0.20** | **0.25** |
| 0.25 | 0.659 | 0.804 | **0.875** | **0.969** |
| 0.30 | 0.628 | 0.778 | **0.854** | **0.962** |
| 0.35 | 0.609 | 0.762 | **0.842** | **0.958** |
| 0.40 | 0.599 | 0.756 | **0.838** | **0.958** |

|  |  | | |
| --- | --- | --- | --- |
|  |  |  |
|  |  |  |  |
|  |  |  |  |
|  |  |  |  |
|  |  |  |  |
|  |  |  |  |
|  |  |  |  |
|  |  |  |  |
|  |  |  |  |
|  |  |  |  |
|  |  |  |  |

Differences of 15% to 20% in viral suppression rates are frequently considered to be evidence of therapeutic superiority in clinical trials comparing antiretroviral regimens. The detectable difference afforded by our sample size is conservative compared to the differences that we estimated in our pilot study. At 6 months and 12 months the absolute differences in viral suppression between DAART and the methadone maintained group who were self-administering HAART were +30 and +23, respectively.

A second factor to consider is the effect size that would be required to make DAART feasible and cost-effective in clinical practice. A recently published paper by Goldie and coworkers [4] explored the economic aspects of adherence-promoting interventions in HIV-infected individuals treated with ART. The authors calculated costs per quality-adjusted life year (QALY) for hypothetical adherence interventions across a wide range of program costs and effect sizes. Effect size was measured in terms of the reduction in the rate of virological failure that was achieved by an intervention. Based on published data, the authors estimated that time- and labor-intensive adherence interventions, like DAART, would cost approximately $500 per person-month. The analysis indicated that such interventions would need to reduce rates of virologic failure by 25% or more to fall below $50,000 per QALY, the customary benchmark for cost-effectiveness. Our observed virologic failure rate in subjects taking self-administered therapy was 75%; a 25% reduction in this failure rate is 56%; 75% minus 56% is an absolute difference of 19%. Thus the Goldie analysis suggests that relatively expensive and intensive adherence interventions, like the one proposed in our study, would need to demonstrate an absolute increase in the viral suppression rate of 20% or more to be cost-effective in clinical practice. Our study is powered to detect an effect size of this magnitude or larger. Thus, based on both the range of effect sizes estimated from our pilot study and cost-effectiveness considerations we believe that our proposed enrollment of 200 individuals will provide adequate statistical power to address our proposed hypotheses.

1. Early stopping rules.

Not applicable

1. **Human Biological Samples, if collected provide information to address all of the following points:**
2. Will samples from living individuals be studied?

Yes

1. Will new samples be obtained and/or will pre-existing samples be studied?

New Samples

1. Will identifiers or codes be retained that could link the identity of the subject to the sample?

Yes

1. Describe procedures to protect against unauthorized use and loss of confidentiality of the samples or inadvertent release of confidential information.

Each study participant will be assigned a unique identification number. This identification number will be used on each data form and clinical specimen to facilitate linkage of data. Names and other obvious identifiers will not be used on forms or clinical specimens. Data collection forms from our pilot study will be modified to create forms tailored for this randomized controlled trial. Study charts will be stored on-site at the methadone clinics in locked file cabinets. The data manager will enter information from paper forms into the electronic database. A programmer will design a Microsoft Access relational database, which will link data from the various sources by unique identifying number. Computers used for data entry will be password protected and data will be backed-up each night.

Clinical specimens (blood and urine samples) will be obtained by the study coordinator at assessment visits, and, in some cases, for study eligibility screening. In addition to the unique identification number, clinical specimens will be further identified by a two digit visit code, a one digit code for specimen type, and a one digit code identifying the specimen aliquot for banked plasma.

**MEMS pilot study**

Participants will be assigned a 3-digit identification number for this study and all MEMS adherence information will be collected and linked to this number only. No personal identifiers will be used to link adherence data to participants.

1. Describe the plans to contact subjects or to access their medical records.

Participant follow-up tracking

In the proposed study we will obtain detailed contact information from participants, including current address, phone number, work number, cell phone number, and pager number. Additionally, we will ask participants to identify and provide contact information for two individuals with whom they have had longstanding relationships (such as family members). Prior to assessment visits, participants who remain in the methadone programs will be given verbal reminders and a written reminder of their upcoming visit. Attempts will be made to locate study participants who are no longer attending the methadone clinic through reminder letters, telephone calls, and personal contacts.

During informed consent, participants will be asked to two release-of-information forms, one for HIV-related medical records, and one for substance abuse treatment records (Appendix).

1. Will specimens be collected for "banking" and future research?

Specimens will be stored for antiretroviral resistance testing only. This will be done to minimize the cost of testing, by testing only the sample that is most likely to show the evolution of new resistance.

**MEMS pilot study**

No samples collected for MEMS pilot study.

1. Describe procedures for obtaining consent for future studies of existing samples.

N/A

1. If genetic testing will be conducted: describe plans for contact of relatives of an existing proband and include any proposed written contact letter or materials.

N/A

1. Describe plans for disclosure of test information including to whom will information be disclosed and by whom.

The following laboratory data will be disclosed to participants’ primary HIV medical providers to assist with medical management – HIV RNA, CD4 cell count.

The following information will not be disclosed to anyone without written permission of the participant – survey data, urine drug screen results

**MEMS pilot study**

Adherence information will be reviewed with participants at the conclusion of the monitoring period. Adherence data will not be shared with medical providers or other clinical staff unless written authorization is provided by the participant.

1. Describe how genetic counseling will be provided prior to and following disclosure

N/A

1. **Risks**
2. Medical risks, listing all procedures, their major and minor risks and expected frequency.

The medical risks of this study are commensurate with the use of antiretroviral therapy. There are currently over 20 licensed antiretroviral drugs and combination preparations, with different adverse event profiles. This clinical trial is designed to assess a behavioral intervention to improve medication adherence and HIV treatment outcomes. It is not a comparison of specific antiretroviral drugs or combinations of drugs. Participation in the study may pose a risk to subject confidentiality, in that staff or other clients at the methadone clinic may become aware of a subject’s HIV status.

**MEMS pilot study**

Medical risks are negligible.

1. Steps taken to minimize the risks.

Participants in both arms of this study will receive monitoring that is likely more intensive than that used in routine clinical care. We believe that this will minimize the overall risk of serious adverse reactions or drug interactions in study subjects.

Antiretroviral drugs will be chosen by participants’ medical providers. All regimens will be reviewed by study coordinators or the PI for potential drug interactions or combinations of drugs that do not conform to current HIV treatment guidelines. Potential interactions with methadone will be communicated to participants, participants’ medical providers, and substance abuse counselors, so that methadone dose adjustments may be anticipated. Study participants will be monitored by study staff for adverse reactions to antiretroviral therapy. Any suspected adverse reactions will be rapidly communicated to participants’ HIV medical providers. Acutely ill participants will be triaged to their medical care provider or an emergency department as appropriate. Laboratory data (CD4+ cell count and HIV RNA) will be performed in real time and results will be sent to participants’ HIV medical providers.

All data collection forms and other records containing participant identifiers will be kept in a locked file cabinet, accessible only to authorized study staff. Computer-based participant surveys will be conducted in a private office with responses stored digitally. Data entered into computerized databases will be password protected. Only authorized study staff will have access to hard-copy or electronic files. All electronic communications involving study data will be encrypted. Study information will not be released without written permission of the subject, except as necessary for monitoring by the Johns Hopkins Institutional Review Board, the NIH, or other governmental regulatory body.

1. Safety monitoring plan including plans for a Data Safety Monitoring Board (DSMB)

This clinical trial compares directly administered antiretroviral therapy (DAART) to standard care (self-administered therapy) in a sample of HIV-infected participants receiving combination antiretroviral therapy and opioid replacement therapy. The behavioral adherence intervention being assessed in our trial is unlikely to pose major safety risks to participants. The study coordinator, site managers, and other study staff will report all potential serious or important adverse events (whether anticipated or unanticipated) to the PI and the participant’s primary medical provider immediately. Unanticipated problems involving risks to participants and others will be reported promptly to the IRB as described in JHM IRB guidelines (<http://irb.jhmi.edu/Policies/103_6b.html>). No DSMB will be used and no interim analyses are planned for this phase II, behavioral clinical trial.

1. For DSMBs, describe details on its operation including membership and reporting procedures.

N/A

1. Plan for reporting adverse events.

Adverse events will be reported to the Johns Hopkins IRB in accordance with the published IRB guidance on this issue (<http://irb.jhmi.edu/Policies/103_6b.html>).

1. Legal risks such as the risks that would be associated with breach of confidentiality.

As this study includes collection of information related to illicit drug use, a potential legal risk could be posed to participants by a breach of confidentiality. In addition to the standard methods of securing data, described above, we have acquired a Certificate of Confidentiality from NIDA to provide further legal protection of the data.

**MEMS pilot study**

Legal risks of the MEMS pilot study are negligible.

1. Financial risks to the subjects.

N/A

1. **Confidentiality**

a. Description of the procedures to be used to protect confidentiality of data collected and stored for research purposes. If sensitive information (illicit drug use, illegal activity, etc.) will be collected, indicate whether a Certificate of Confidentiality would be obtained (See the JHM-IRB Guidelines for information on how to obtain a Certificate).

Each study participant will be assigned a unique identification number. This identification number will be used on each data form and clinical specimen to facilitate linkage of data. Names and other obvious identifiers will not be used on forms or clinical specimens. Data collection forms from our pilot study will be modified to create forms tailored for this randomized controlled trial. Study charts will be stored on-site at the methadone clinics in locked file cabinets. The data manager will enter information from paper forms into the electronic database. A programmer will design a Microsoft Access relational database, which will link data from the various sources by unique identifying number. Computers used for data entry will be password protected and data will be backed-up each night.

Clinical specimens (blood and urine samples) will be obtained by the study coordinator at assessment visits. In addition to the unique identification number, clinical specimens will be further identified by a two digit visit code, a one digit code for specimen type, and a one digit code identifying the specimen aliquot for banked plasma.

All data collection forms and other records containing participant identifiers will be kept in a locked file cabinet, accessible only to authorized study staff. Computer-based participant surveys will be conducted in a private office with responses stored digitally. Data entered into computerized databases will be password protected. Only authorized study staff will have access to hard-copy or electronic files. All electronic communications involving study data will be encrypted. Study information will not be released without written permission of the subject, except as necessary for monitoring by the Johns Hopkins Institutional Review Board, the NIH, or other governmental regulatory body.

In addition to the standard methods of securing data, described above, we have obtained a Certificate of Confidentiality from NIDA to provide further legal protection of the data.

**MEMS pilot study**

Participants will be assigned a 3-digit identification number for this study and all MEMS adherence information will be collected and linked to this number only. No personal identifiers will be used to link adherence data to participants.

1. **Benefits**
2. Description of the probable benefits.

i. Individual subject.

Improved retention to and adherence with antiretroviral therapy

ii. Society.

If DAART found to be an efficacious adherence intervention for HIV-infected drug users receiving methadone.

**MEMS pilot study**

i. MEMS monitoring may help participant identify adherence problems he or she is having

ii. None

12. **Compensation**

a. Detail compensation for subjects including possible total compensation, proposed bonus, and any proposed reductions or penalties for not completing the protocol, free testing.

Subjects will be given $50 for each of the 5 study visits that they complete (total $250 for completing all study visits). The study visits are at baseline, 3 months, 6 months, 12 months, and 18 months. Subjects will be also be given $20 at both the MEMS instructional visit, and at the 1.5-week follow-up visit. They will also be reimbursed $30 at both the 4-week and 8-week follow-up MEMS visits (total $100 for completing MEMS monitoring phase). The total amount subjects could receive in this 18-month study is $350 if they complete all of the study visits and return the MEMS cap when requested.

**MEMS pilot study**

Participants in the MEMS pilot who return their MEMS caps for data downloads will be compensated $10 at both the 1-week and 2-week follow-up, and $20 at the 4-week follow-up visit (maximum compensation: $40).

13 **Costs**

1. Detail costs of study procedure(s) or drug(s) or substance(s) to subjects and identify who will pay for them

N/A

1. **Consent**
2. Identify Type of Consent

i. Written informed consent form(s) to be signed by all subjects (complete Form B and C for each consent form) in the main study and in the MEMS pilot study.

1. Payment for study subjects.

Subjects will be given $50 for each of the 5 study visits that they complete (total $250 for completing all study visits). The study visits are at baseline, 3 months, 6 months, 12 months, and 18 months. Subjects will be also be given $20 at both the MEMS instructional visit, and at the 1.5-week follow-up visit. They will also be reimbursed $30 at both the 4-week and 8-week follow-up MEMS visits (total $100 for completing MEMS monitoring phase). The total amount subjects could receive in this 18-month study is $350 if they complete all of the study visits and return the MEMS cap when requested.

**MEMS pilot study**

Participants in the MEMS pilot who return their MEMS caps for data downloads will be compensated $10 at both the 1-week and 2-week follow-up, and $20 at the 4-week follow-up visit (maximum compensation: $40).

1. Persons conducting the informed consent discussion with the subject.

Informed consent discussions for this study will be performed the three site managers at the study sites and by the Study Coordinator. All of these individuals has completed on-line courses and passed the follow-up exams required to conduct informed consent discussions, either at Johns Hopkins or at their own institution.

**MEMS pilot study**

Informed consent will be performed by either B. Anna Mullen (study coordinator) or Tracey Whoolery (site manager at PAODD).

1. Where and when will consent be obtained.

At the candidate’s OTP in a private room.

1. Time allotted for obtaining consent.

40 minutes

1. Reading level of consent form (indicate software program used to determine).

Flesh-Kincaid reading level 8.5, Microsoft Word feature.

1. Describe how comprehension of the consent information will be assessed.

After the consent document has been presented to a participant, the consent designee will ask him or her whether they have any questions. If the participant does not, the consent designee will ask him or her to briefly describe what will happen in this study. The study coordinator will also ask closed ended questions, such as “What are the two types of treatment in this study”, and “How do we decide which type of treatment you will get”

1. Describe how capacity for consent will be determined if some or all subjects are cognitively impaired or have language/hearing impairment. Describe the procedure for identifying legal representatives for those unable to consent.

The ability of a participant to understand the study will be assessed with the questions described in (g) above. Misconceptions about the study will be corrected. Individuals who are incapable of providing informed consent will not be enrolled.

1. **Consent Form including all of the following applicable paragraph headings**.
2. Purpose*
3. Procedures*
4. Withdrawal Procedures
5. Subject Termination
6. Risks/Discomforts*
7. Pregnancy Risks
8. Benefits*
9. Costs
10. Compensation
11. Alternatives*

***Required elements**

**Form B**

**Main Study**

**Verify that the informed consent document contains each of the eight required elements (45 CFR 46.116):**

| **YES** | **NO** | **REQUIRED ELEMENT** |
| --- | --- | --- |
| X | __ | 1. A statement that the study involves research, an explanation of the purposes of the research and the expected duration of the subject’s participation, a description of the procedures to be followed, and identification of any procedures which are experimental. |
| X | __ | 2. A description of any reasonably foreseeable risks or discomforts to the subject. |
| X | __ | 3. A description of any benefits to the subject or others which may reasonably be expected from the research. |
| X | __ | 4. A statement of appropriate alternative procedures or courses of treatment, if any, that might be advantageous to the subject. |
| X | __ | 5. A statement describing the extent, if any, to which confidentiality of records identifying the subject will be maintained. |
| X | __ | 6. For research involving more than minimal risk, an explanation as to whether any compensation and an explanation as to whether any medical treatments are available if injury occurs and, if so, what they consist of, or where further information may be obtained. |
| X | __ | 7. An explanation of whom to contact for answers to pertinent questions about the research and research subjects’ rights, and whom to contact in the event of a research-related injury to the subjects. |
| X | __ | 8. A statement that the participation is voluntary, refusal to participate will involve no penalty or loss of benefits to which the subject is otherwise entitled, and the subject may discontinue participation at any time without penalty or loss of benefits to which the subject is otherwise entitled. |

**Form C**

**Main Study**

**When appropriate, which of the following additional elements are provided in the consent form?**

| **YES** | **NO** | **N/A** | **ADDITIONAL ELEMENTS** |
| --- | --- | --- | --- |
| X | ___ | ___ | 1. A statement that the particular treatment or procedure may involve risks to the subject (or to the embryo or fetus, if the subject is or may become pregnant) which are currently unforeseeable. |
| X | ___ | ___ | 2. Anticipated circumstances under which the subject’s participation may be terminated by the investigator without regard to the subject’s consent. |
| ___ | ___ | _X | 3. Any additional costs to the subject that may result from participation in the research. |
| X | ___ | ___ | 4. The consequences of a subject’s decision to withdraw from the research and the procedures for orderly termination of participation by the subject. |
| ___ | ___ | X_ | 5. A statement that significant new findings developed during the course of the research which may relate to the subject’s willingness to continue participation will be provided to the subject. |
| X | ___ | ___ | 6. The approximate number of subjects involved in the study. |

**Form B**

**MEMS Pilot study**

**Verify that the informed consent document contains each of the eight required elements (45 CFR 46.116):**

| **YES** | **NO** | **REQUIRED ELEMENT** |
| --- | --- | --- |
| X | __ | 1. A statement that the study involves research, an explanation of the purposes of the research and the expected duration of the subject’s participation, a description of the procedures to be followed, and identification of any procedures which are experimental. |
| X | __ | 2. A description of any reasonably foreseeable risks or discomforts to the subject. |
| X | __ | 3. A description of any benefits to the subject or others which may reasonably be expected from the research. |
| X | __ | 4. A statement of appropriate alternative procedures or courses of treatment, if any, that might be advantageous to the subject. |
| X | __ | 5. A statement describing the extent, if any, to which confidentiality of records identifying the subject will be maintained. |
| X | __ | 6. For research involving more than minimal risk, an explanation as to whether any compensation and an explanation as to whether any medical treatments are available if injury occurs and, if so, what they consist of, or where further information may be obtained. |
| X | __ | 7. An explanation of whom to contact for answers to pertinent questions about the research and research subjects’ rights, and whom to contact in the event of a research-related injury to the subjects. |
| X | __ | 8. A statement that the participation is voluntary, refusal to participate will involve no penalty or loss of benefits to which the subject is otherwise entitled, and the subject may discontinue participation at any time without penalty or loss of benefits to which the subject is otherwise entitled. |

**Form C**

**MEMS pilot study**

**When appropriate, which of the following additional elements are provided in the consent form?**

| **YES** | **NO** | **N/A** | **ADDITIONAL ELEMENTS** |
| --- | --- | --- | --- |
| X | ___ | ___ | 1. A statement that the particular treatment or procedure may involve risks to the subject (or to the embryo or fetus, if the subject is or may become pregnant) which are currently unforeseeable. |
| X | ___ | ___ | 2. Anticipated circumstances under which the subject’s participation may be terminated by the investigator without regard to the subject’s consent. |
| ___ | ___ | X_ | 3. Any additional costs to the subject that may result from participation in the research. |
| X | ___ | ___ | 4. The consequences of a subject’s decision to withdraw from the research and the procedures for orderly termination of participation by the subject. |
| ___ | ___ | X_ | 5. A statement that significant new findings developed during the course of the research which may relate to the subject’s willingness to continue participation will be provided to the subject. |
| X | ___ | ___ | 6. The approximate number of subjects involved in the study. |
